# Adaptive hydrogel platforms for infected bone defect repair: microenvironmental responsiveness, osteoimmunomodulation, and regenerative remodeling

**DOI:** 10.3389/fbioe.2026.1932272

**Published:** 2026-09-24

**Authors:** Jianbo Feng, Huang Kun, Shiping Chen, Fang Chen, Yunbo Ding, Qiang Zhang, Guangjin Feng, Jiahao Xiong, Yanyang Chen, Jinglin Li

**Affiliations:** 1 Department of Orthopedics, The People’s Hospital of Zheng’an, Zunyi, China; 2 Department of Orthopedics, Affiliated Hospital of Zunyi Medical University, Zunyi, China; 3 The First People’s Hospital of Zunyi, Zunyi, China; 4 Joint Orthopaedic Research Center of Zunyi Medical University, University of Rochester Medical Center, Zunyi, China; 5 Department of Orthopedics, The First Affiliated Hospital of Bengbu Medical University, Bengbu, China

**Keywords:** adaptive hydrogel, antibacterial therapy, bone regeneration, infected bone defect, macrophage polarization, microenvironmental responsiveness, osteoimmunomodulation

## Abstract

Infected bone defects remain a major clinical challenge because persistent bacterial colonization and biofilm formation coexist with chronic inflammation, oxidative stress, osteoimmune imbalance, vascular injury, and impaired bone regeneration. Conventional treatment relies primarily on surgical debridement, antimicrobial therapy, and bone reconstruction, but often fails to simultaneously eradicate biofilms, maintain effective local drug concentrations, restore the inflammatory microenvironment, and support structural bone repair. Hydrogels have emerged as promising local therapeutic platforms owing to their injectability, defect-filling capacity, local retention, controllable degradation, and cargo-loading versatility. Stimuli-responsive hydrogel modules can react to endogenous pathological cues, including acidic pH, elevated reactive oxygen species, abnormal enzyme activity, and hypoxia, whereas near-infrared light and related physical inputs provide externally triggered control. By integrating responsive modules with multi-cue recognition, lesion-state-dependent release, or coordinated functional regulation, adaptive hydrogel platforms may couple antibacterial and antibiofilm activity with immune remodeling, vascularization, and osteogenic regeneration. This review summarizes the pathological microenvironment of infected bone defects and recent advances in responsive and adaptive hydrogel systems. Particular attention is given to macrophage-mediated osteoimmunomodulation and the regulation of macrophage functional states beyond a fixed M1/M2 framework. Antibacterial defense, inflammation resolution, vascular reconstruction, and regenerative repair are interpreted as overlapping functional requirements rather than universally discrete chronological stages, with their relative importance changing according to bacterial burden, biofilm activity, oxidative and inflammatory stress, tissue perfusion, and regenerative capacity. We further evaluate the integration of antibacterial, immunomodulatory, angiogenic, and osteogenic modules, distinguish direct infected-bone-defect evidence from extrapolated findings, and discuss experimental models, operational criteria for adaptive switching, manufacturing complexity, biosafety, and translational challenges. This framework may guide the development of hydrogel platforms that more precisely match therapeutic functions to the evolving state of infected bone defects.

## 1 Introduction

Infected bone defects represent a particularly challenging condition in orthopedic practice. They commonly arise from open fractures, post-traumatic infection, chronic osteomyelitis, implant-associated infection, diabetic foot-related bone infection, and bone loss following tumor resection or surgical debridement. Unlike aseptic bone defects, they are characterized by the coexistence of persistent microbial colonization, structural bone destruction, dysregulated inflammation, and impaired regeneration. Their management therefore requires both infection control and restoration of bone structure and function (Ma L. et al., 2024; Masters et al., 2019; Yu et al., 2026a). *Staphylococcus aureus*, methicillin-resistant *Staphylococcus aureus* (MRSA), *Staphylococcus epidermidis*, and certain Gram-negative bacteria can persist on necrotic bone, implant surfaces, and defect margins. Biofilm formation further protects these pathogens from antimicrobial agents and host immune clearance, contributing to recurrent infection, chronic inflammation, and failed bone repair (Yu et al., 2026b; Rozis et al., 2021). Infected bone defects should therefore be viewed as a complex pathological ecosystem involving bacteria, biofilms, immune cells, bone-resident cells, vascular injury, and the local biomaterial environment (Zhang R. et al., 2026; Zimmerli and Sendi, 2017).

Current treatment relies primarily on radical debridement, systemic or local antibiotics, bone grafting, bone transport, and internal or external fixation. Although clinically indispensable, these approaches have important limitations (Ying et al., 2025). Impaired perfusion, necrotic bone, and biofilm formation restrict antibiotic penetration and make it difficult to maintain effective local drug concentrations. Biofilm-associated bacteria also exhibit reduced metabolic activity and enhanced tolerance to antimicrobial therapy, increasing the risks of recurrence and resistance. Moreover, bacterial clearance alone does not restore the regenerative capacity of infected bone tissue (Sharif and Yadav, 2025; Yuan et al., 2024). Persistent inflammatory cytokine production, oxidative stress, osteoclast activation, vascular dysfunction, impaired osteoblast activity, and reduced osteogenic differentiation of bone marrow mesenchymal stem cells collectively maintain a microenvironment unfavorable for repair (Li J. et al., 2026). Effective local therapy must therefore address infection, biofilms, host-tissue injury, immune dysregulation, and bone regeneration in an integrated manner.

Hydrogels have attracted increasing attention as local therapeutic platforms because of their biocompatibility, hydrated structure, injectability, defect-filling ability, local retention, controllable degradation, and cargo-loading versatility (Huang L. et al., 2025). They can deliver antibiotics, antimicrobial peptides, metal ions, nanomaterials, growth factors, exosomes, and immunomodulatory agents directly to irregular defect sites, thereby increasing local exposure while reducing systemic toxicity (Wang C. et al., 2023). However, sustained release through diffusion, swelling, or degradation does not by itself constitute adaptability. In this review, the relevant terms are used hierarchically. “Stimuli-responsive” refers to a predefined material response to an endogenous or exogenous stimulus. “Microenvironment-responsive” is restricted to systems activated by lesion-associated cues such as acidic pH, elevated reactive oxygen species, abnormal enzyme activity, or hypoxia, whereas systems activated by near-infrared light or other externally applied physical inputs are described as externally triggered. “Multifunctional” denotes the incorporation of multiple therapeutic functions, and “sequential-release” denotes the ordered delivery of different cargos; neither property alone necessarily defines an adaptive system. Here, “adaptive hydrogel platforms” refers to higher-order systems that integrate responsive modules, temporal programming, lesion-state-dependent release, or feedback-related regulation to adjust antibacterial, immunomodulatory, angiogenic, and osteogenic functions according to changing pathological requirements. Because most reported systems have not achieved fully autonomous closed-loop control, adaptability is used as an operational system-level concept rather than as evidence of complete biological autonomy (Salama et al., 2025; Xie et al., 2025; Yu et al., 2024; Chen Y. et al., 2025).

Osteoimmune regulation is a central component of this framework. Macrophages participate in pathogen recognition and phagocytosis, inflammatory amplification and resolution, angiogenesis, osteoclast regulation, and osteogenic repair. Although macrophages have traditionally been classified into pro-inflammatory M1 and anti-inflammatory or reparative M2 phenotypes, this binary model does not adequately reflect their functional plasticity in infected bone defects (Li et al., 2025a). Strong antibacterial and phagocytic activity may be required while bacterial burden remains high, whereas persistent inflammatory activation after infection pressure decreases can aggravate tissue injury and impair regeneration. Macrophage regulation should therefore be understood as lesion-state-dependent remodeling of functional programs rather than unidirectional suppression of M1 polarization or promotion of a fixed M2 phenotype. Hydrogels can influence these programs through their physicochemical properties and through localized delivery of antibacterial, antioxidant, metabolic, and immunomodulatory components, making macrophages important links between infection control and regenerative remodeling (Shi et al., 2025).

Because direct studies of adaptive hydrogels in infected bone defects remain limited, this review also includes selected evidence from adjacent disease models and related material systems. The evidence is interpreted at three levels. Direct disease-specific evidence refers to hydrogel studies conducted in infected bone defect or osteomyelitis models that assess infection control together with bone-regeneration outcomes. Partially relevant evidence includes bone infection, implant-associated infection, periodontitis, biofilm, or infected soft-tissue wound studies that evaluate antibacterial or immunomodulatory effects without fully reproducing structural infected bone defects. Studies involving osteoarthritis, osteoporosis, aseptic bone defects, diabetic or burn wounds, tumor models, coatings, alloys, nanoparticles, and other non-hydrogel systems are considered extrapolated mechanistic or material-design evidence rather than direct validation. On this basis, the review examines the pathological microenvironment of infected bone defects; summarizes endogenous cue-responsive and externally triggered hydrogel strategies; discusses antibacterial, antibiofilm, and macrophage-mediated osteoimmune regulation; and evaluates multifunctional integration, experimental models, operational criteria for adaptive switching, and translational challenges. The aim is to provide a critical framework for designing hydrogel platforms whose therapeutic functions are matched to the evolving biological state of infected bone defects.

## 2 Pathological microenvironment of infected bone defects

### 2.1 Bacterial colonization and biofilm formation: a central barrier to infection persistence

The difficulty in repairing infected bone defects lies not only in the loss of bone structure but also in the coexistence of multiple pathological alterations, including persistent bacterial colonization, biofilm formation, sustained inflammation, enhanced oxidative stress, impaired vascular supply, immune cell dysfunction, and disruption of the osteogenesis–osteoclastogenesis balance (Ma et al., 2025). Persistent bacterial colonization is a key initiating factor that drives the development and chronic progression of infected bone defects. *Staphylococcus aureus*, particularly methicillin-resistant *S. aureus* (MRSA), is among the most common pathogens associated with bone infection and infected bone defects. In addition, *S. epidermidis*, *Escherichia coli*, *Pseudomonas aeruginosa*, and polymicrobial infections can also contribute to disease progression (Seebach et al., 2023; Xu et al., 2025; Duan et al., 2025). These pathogens can adhere to necrotic bone, bone defect surfaces, implants, or local scaffold materials, and adapt to the bone microenvironment by expressing adhesion factors, secreting virulence factors, and altering their metabolic state. Biofilm formation is one of the major reasons why infected bone defects are difficult to eradicate (Zhang R. et al., 2025). Biofilms consist of bacterial communities embedded within self-produced extracellular polymeric substances, which encapsulate bacteria and create a relatively closed microbial ecosystem. Compared with planktonic bacteria, bacteria within biofilms exhibit markedly increased tolerance to both antibiotics and host immune attack (Masters et al., 2022). On the one hand, the extracellular polymeric matrix limits the penetration of antimicrobial agents into the deeper layers of biofilms; on the other hand, bacteria residing inside biofilms often enter a low-metabolic or dormant-like state, rendering them less susceptible to antibiotics that depend on active bacterial proliferation (Cella et al., 2026). Moreover, biofilms impair the phagocytic and clearance capacities of neutrophils and macrophages, thereby promoting long-term infection persistence. In infected bone defects, biofilms act not only as physical barriers to antimicrobial therapy but also as important sources of chronic inflammation and immune dysregulation (Evans et al., 2025). Persistent bacteria and their virulence factors continuously activate local immune cells and induce the release of inflammatory cytokines such as TNF-α, IL-1β, and IL-6, further aggravating bone destruction (Li et al., 2025a). Therefore, treatment strategies for infected bone defects should not be limited to eliminating planktonic bacteria; they should also disrupt mature biofilms, inhibit biofilm re-formation, and restore the host immune system’s capacity to clear pathogens.

### 2.2 Sustained inflammation and oxidative stress: key drivers of tissue damage and impaired bone repair

Compared with aseptic bone defects, infected bone defects are characterized by a highly dynamic and mutually reinforcing pathological microenvironment. Pathogens and their virulence factors induce persistent inflammatory responses; cytokines and reactive oxygen species released by inflammatory cells further exacerbate tissue injury; and bone destruction, sequestrum formation, and vascular impairment create favorable conditions for bacterial survival and biofilm development (Zhang et al., 2025b). During the early stage of infection, a moderate inflammatory response facilitates pathogen recognition, immune cell recruitment, and bacterial clearance. However, when infection is not controlled in a timely manner, inflammation shifts from a protective response to a persistent driver of tissue damage. Infected bone defects are typically accompanied by infiltration of neutrophils, macrophages, dendritic cells, and other immune cells. After recognizing bacteria-associated molecular patterns, these cells release large amounts of pro-inflammatory cytokines, chemokines, and reactive oxygen species. As central effector cells in infected lesions, macrophages exhibit high plasticity and can be polarized into pro-inflammatory M1-like or anti-inflammatory/reparative M2-like phenotypes in response to microenvironmental cues. Under pathological conditions of infected bone defects, persistent bacteria and biofilms may maintain macrophages in a prolonged M1-like inflammatory state, thereby disturbing the normal transition from early inflammatory activation to reparative immune remodeling required for bone healing. This sustained inflammatory state suppresses osteogenesis while promoting bone resorption (Li et al., 2025a; Wang et al., 2025). Persistently elevated pro-inflammatory cytokines can directly disrupt bone remodeling through multiple mechanisms. TNF-α and IL-1β can synergistically activate NF-κB and MAPK signaling pathways and upregulate RANKL expression in osteoblasts and stromal cells. Binding of RANKL to RANK on osteoclast precursors subsequently activates downstream transcription factors such as NFATc1, driving osteoclast differentiation and activation and leading to enhanced bone resorption (Johnson et al., 2019; Mussbacher et al., 2023). Inflammatory cytokines can also impair local vascular endothelial cell function, disrupt microcirculatory stability, and further reduce nutrient and oxygen supply within the bone defect region (Xia et al., 2025). Oxidative stress plays a dual role in infected bone defects. Physiological levels of reactive oxygen species are involved in pathogen killing and constitute an important component of the antibacterial responses of neutrophils and macrophages. However, prolonged and excessive accumulation of reactive oxygen species can damage host tissues and amplify inflammatory responses. Excessive reactive oxygen species induce lipid peroxidation, mitochondrial dysfunction, and protein and DNA damage, ultimately leading to impaired function or death of osteogenesis-related cells. Evidence from periodontal bone regeneration, aged bone-defect, and diabetic wound models further suggests that excessive oxidative stress can impair osteogenic cells and prolong pro-inflammatory macrophage activation. These findings provide mechanistic support for the detrimental role of ROS, but do not constitute direct validation in infected bone defects (Yu et al., 2026c; Sun X. et al., 2026; Sun C. et al., 2026). Therefore, limiting excessive oxidative injury while preserving the necessary antibacterial activity of reactive oxygen species remains a key challenge in the repair of infected bone defects.

### 2.3 Acidic, hypoxic, and enzyme-abnormal microenvironments: pathological signals for responsive material design

The local microenvironment of infected bone defects is commonly characterized by acidic pH, elevated reactive oxygen species (ROS), hypoxia, and abnormal expression of multiple enzymes. These pathological signals not only contribute to disease progression but also provide important targets for the microenvironment-responsive design of adaptive hydrogels (Yu et al., 2026b; Li X. et al., 2026; Kong et al., 2025). Local acidification frequently occurs in infected and inflamed tissues. Bacterial metabolites, enhanced glycolysis in inflammatory cells, insufficient local blood supply, and tissue necrosis can all contribute to the formation of an acidic microenvironment (Xie et al., 2025). Such acidic conditions may favor the survival of certain pathogens and affect antibiotic activity, cellular metabolism, and bone matrix mineralization. From a materials design perspective, acidic pH can serve as a triggering signal for pH-responsive hydrogels, enabling lesion-specific release of antibacterial drugs, metal ions, or immunomodulatory agents (Chen et al., 2026; Chen H. et al., 2023). Marked hypoxia is another common feature of infected bone defects. Bone infection can cause local vascular injury, microcirculatory dysfunction, and tissue edema, while the bone defect area after debridement often suffers from insufficient vascular reconstruction. Hypoxia affects immune cell metabolism and functional states and regulates inflammatory responses, angiogenesis, and bone regeneration through HIF-related pathways (Weng et al., 2026). In infected bone defects, hypoxia exerts stage-dependent and context-specific effects. Moderate hypoxia can induce the expression of angiogenesis-related factors through the HIF-1α/VEGF axis and serves as an important physiological signal for initiating early vascularization during bone repair. However, when hypoxia becomes severe or persistent, excessive HIF-1α activation may inhibit osteoblast proliferation by suppressing the Wnt pathway and osterix expression (Lee et al., 2024). In addition, crosstalk between HIF-1α and NF-κB signaling under hypoxic conditions may promote M1-like macrophage polarization, further amplifying local inflammatory responses (Li J. et al., 2026). Therefore, alleviating pathological hypoxia, promoting vascularization, and restoring nutrient supply are critical steps in the regenerative repair of infected bone defects. A variety of bacteria-derived enzymes and host-derived inflammation-associated enzymes are also present in infected lesions. *Staphylococcus aureus* can secrete multiple extracellular enzymes, including nucleases, proteases, lipases, hyaluronidases, and collagenases. These enzymes degrade host tissues to provide nutrients for bacterial survival and facilitate tissue invasion by disrupting intercellular junctions and extracellular matrix components (Shettigar and Murali, 2020; Campbell et al., 2023). In inflamed tissues, matrix metalloproteinases and other degradative enzymes are often abnormally upregulated and participate in extracellular matrix destruction and tissue remodeling (Cui et al., 2017). These enzymatic abnormalities not only aggravate bone and soft tissue damage but can also be exploited as triggering cues for enzyme-responsive hydrogels, enabling localized degradation or controlled drug release (Zhang et al., 2024). Collectively, acidic pH, elevated ROS, hypoxia, and abnormal enzyme activity constitute major microenvironmental features that distinguish infected bone defects from ordinary bone defects. Adaptive hydrogels designed to respond to these pathological signals may therefore achieve lesion-localized release, inflammatory microenvironment regulation, and stage-specific tissue repair.

### 2.4 Osteoimmune imbalance: aberrant interactions among macrophages, osteoclasts, and osteoblast-lineage cells

Bone repair depends on the precise coordination between the immune system and bone cell populations. In infected bone defects, immune responses often remain abnormally activated for prolonged periods, resulting in osteoimmune microenvironmental imbalance (Liu et al., 2022). Macrophages are among the central cellular mediators of this process, as their functional states directly influence pathogen clearance, inflammation resolution, angiogenesis, and bone regeneration. During the early stage of infection, macrophages contribute to antibacterial defense by phagocytosing bacteria, releasing inflammatory mediators, and generating ROS. Appropriate pro-inflammatory activation is beneficial for infection control. However, when bacteria and biofilms persist, macrophages may remain in a sustained pro-inflammatory state and continuously secrete inflammatory mediators such as TNF-α, IL-1β, and IL-6. These mediators not only aggravate local tissue injury but also promote RANKL expression, thereby enhancing osteoclastogenesis and bone resorption (Zhang et al., 2025a). Meanwhile, a persistent inflammatory environment suppresses osteoblast activity and the osteogenic differentiation of bone marrow mesenchymal stem cells (BMSCs), ultimately delaying or arresting bone repair. Compared with aseptic bone defects, macrophage regulation in infected bone defects is more complex. The traditional M1/M2 dichotomy is useful for understanding the differences between pro-inflammatory and reparative macrophage states, but it cannot fully explain the dynamic immune requirements during infection-associated bone repair. In the early phase of infection, a certain degree of antibacterial immune activation is required for pathogen clearance. After infection is gradually controlled, the local immune environment should shift toward inflammation resolution, angiogenesis, and tissue repair. Premature or excessive suppression of inflammation may impair pathogen clearance, whereas persistent and excessive pro-inflammatory responses can aggravate bone resorption and impair osteogenesis (Shi et al., 2025). Therefore, infected bone defects require stage-specific and dynamic regulation of macrophage functions rather than simple promotion or inhibition of a single macrophage phenotype. In addition to macrophages, osteoclasts and osteoblast-lineage cells are also important participants in osteoimmune imbalance. Inflammatory cytokines, bacterial virulence factors, and oxidative stress can all promote osteoclast differentiation and bone resorption. At the molecular level, *S. aureus* infection can strongly stimulate RANKL upregulation in bone marrow stromal cells and disrupt the RANKL/OPG balance through multiple virulence factors, thereby driving excessive osteoclast activation (Beenken et al., 2026). In contrast, osteoblasts are subjected to dual injury from inflammatory mediators and bacterial virulence factors within the infected microenvironment. Staphylococcal protein A (SpA), a major virulence factor of *S. aureus*, can directly bind to osteoblasts through TNFR1 and activate intracellular apoptotic signaling cascades. This leads to reduced osteoblast proliferation, increased apoptosis, and impaired alkaline phosphatase activity, type I collagen synthesis, and mineralized nodule formation. Meanwhile, infected osteoblasts themselves can upregulate RANKL expression, further promoting osteoclastogenesis (Granata et al., 2022; Marriott, 2013; Sanchez et al., 2013). Impaired osteoblast and BMSC function further weakens bone matrix formation, making effective filling and reconstruction of the bone defect difficult to achieve. Taken together, osteoimmune imbalance in infected bone defects is not the result of dysfunction in a single immune cell type. Rather, it reflects the combined consequences of aberrant interactions among macrophages, osteoclasts, osteoblasts, BMSCs, vascular endothelial cells, and the infected local microenvironment.

### 2.5 Impaired osteogenic regeneration and insufficient vascularization: repair bottlenecks after infection control

The ultimate goal of treating infected bone defects is not merely to control infection, but to restore the structure and function of bone tissue. However, even after infection has been partially controlled, local bone regeneration is often constrained by multiple factors. Surgical debridement inevitably removes necrotic bone and infected tissue, frequently resulting in large bone defects. Epidemiological data indicate that the incidence of post-traumatic osteomyelitis ranges from 4.5% to 20%. When repeated debridement fails to control infection, radical removal of infected bone becomes necessary; however, this approach often creates critical-sized or even supercritical bone defects that are unable to heal spontaneously, thereby posing dual challenges of infection control and subsequent reconstruction (Qv et al., 2025). The vascular system provides a structural template for bone development and delivers essential components required for bone homeostasis, including minerals, growth factors, and osteoprogenitor cells, to the osteogenic microenvironment. Vascular endothelial growth factor (VEGF) is a master regulator of vascular growth and is indispensable for the effective coupling of angiogenesis and osteogenesis during skeletal development and postnatal bone repair (Burger et al., 2022). In infected bone defects, infection and inflammation disrupt the local microcirculation, resulting in insufficient neovascularization and restricting the entry of oxygen, nutrients, and reparative cells into the defect region. Chronic inflammation and oxidative stress further suppress the osteogenic activity of osteoblasts and bone marrow mesenchymal stem cells (BMSCs), thereby impairing bone matrix deposition and mineralization (Sun H. et al., 2023). Insufficient vascularization is one of the major causes of regenerative failure in infected bone defects. Fundamentally, bone is a highly vascularized tissue, and bone repair requires the vascular system to coordinate multiple functions, including the supply of oxygen, nutrients, and minerals, as well as the recruitment of immune cells and circulating osteoprogenitor cells into the defect region (Li et al., 2023). In addition, vascular endothelial cells exert “angiocrine” functions by secreting osteogenic growth factors such as BMP-2 and BMP-4, which directly promote osteoblast differentiation and mineralization and thereby support vascularized bone regeneration (Cao H. et al., 2024). In the infected microenvironment, inflammatory injury, thrombosis, tissue necrosis, and hypoxia disrupt the vascular network and leave the defect region in a prolonged hypoperfused state. Even in non-infected critical-sized bone defects, spontaneous angiogenesis is often too slow to establish a dense and functional vascular network throughout the entire defect region in time (Jang and Yoon, 2024). This problem becomes even more pronounced when infection further damages local vessels. Without effective vascular reconstruction, stable new bone formation remains difficult to achieve, even when osteogenic scaffolds or osteoinductive factors are locally implanted. Osteogenic repair in infected bone defects is also limited by an imbalance between osteoclast-mediated bone resorption and osteoblast-mediated bone formation. Persistent inflammation enhances osteoclast activity and accelerates bone resorption, while simultaneously suppressing the expression of osteogenesis-related molecules such as RUNX2, ALP, COL1A1, and OCN, thereby reducing bone matrix formation and mineralization capacity (Kang et al., 2026; Zhong et al., 2026). Therefore, an ideal therapeutic strategy should go beyond infection control by further promoting vascularization, restoring osteoblast function, regulating the balance between bone resorption and bone formation, and guiding long-term bone matrix remodeling (Figure 1). The pathological features summarized above should not be regarded as isolated abnormalities. Infected bone defects generally evolve through an interconnected process in which bacterial colonization and biofilm formation sustain inflammatory and oxidative stress; persistent inflammation and vascular injury subsequently aggravate acidification, hypoxia, immune dysfunction, and osteoclast activation; and these changes ultimately suppress angiogenesis, osteogenic differentiation, and bone matrix reconstruction. These pathological processes do not occur as universally discrete chronological stages. In chronic osteomyelitis and implant-associated infection, bacterial persistence, biofilm formation, inflammatory activation, vascular injury, and attempted tissue repair frequently coexist. Incomplete bacterial clearance or biofilm reactivation may also return the lesion to an infection-dominant state. Therefore, the terms early, intermediate, and late are used in this review to indicate shifts in dominant therapeutic priorities rather than fixed temporal boundaries. When viable bacteria and biofilm activity remain high, antibacterial and antibiofilm functions should predominate. As infection-related pressure decreases but excessive inflammatory and oxidative injury persists, immune-remodeling functions should gain greater importance. When infection control is sustained and vascular or osteogenic insufficiency becomes the principal barrier, angiogenic and osteogenic support should be strengthened. These functions may overlap throughout the repair process and should be rebalanced according to lesion status.

**FIGURE 1:**
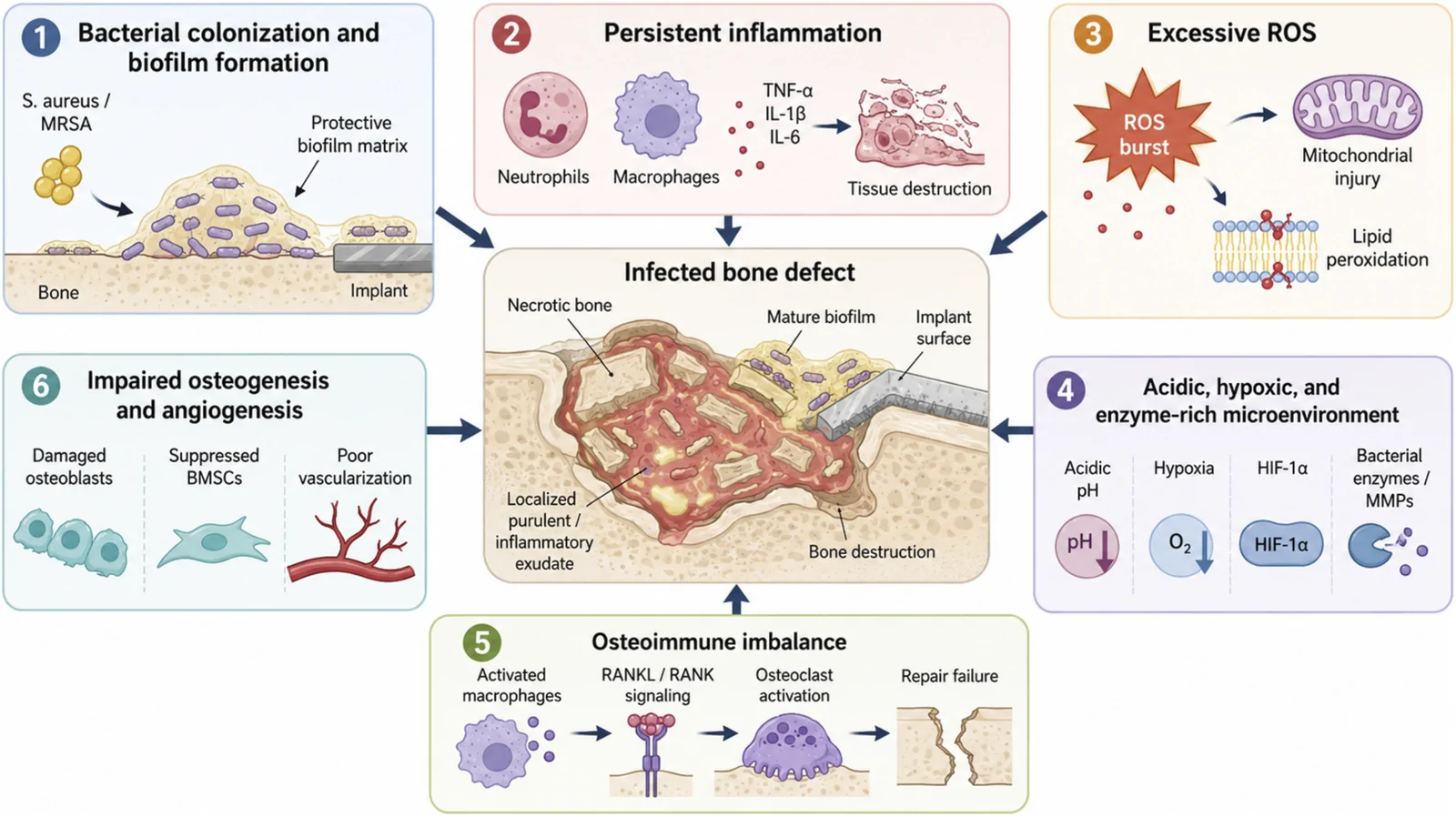
Interconnected pathological microenvironment and evolving repair barriers in infected bone defects. Persistent bacterial colonization and biofilm formation initiate and maintain inflammatory and oxidative stress, while vascular injury, hypoxia, abnormal enzyme activity, and local acidification further impair immune regulation and tissue metabolism. These interacting processes promote osteoclast-mediated bone resorption, suppress osteoblast and BMSC function, and limit vascularized bone regeneration. The elements shown should therefore be interpreted as an interconnected pathological network rather than as independent abnormalities.

## 3 Responsive building blocks and adaptive system design for infected bone defect repair

The construction of adaptive hydrogel platforms generally begins with individual stimulus-responsive building blocks. These responsive modules can be divided into endogenous pathological cue-responsive systems and exogenous stimulus-responsive systems. Endogenous systems are activated by abnormalities within infected lesions, including acidic pH, elevated ROS, abnormal enzyme activity, hypoxia, and biofilm-associated signals. Exogenous systems, particularly photothermal and photodynamic platforms, are activated by externally applied light and provide additional temporal control. A hydrogel responding to only one predefined stimulus is therefore described in this review as a responsive hydrogel rather than a fully adaptive system. Importantly, prolonged release, multiple loaded components, or a predefined sequential-release profile alone does not establish adaptability unless these features are explicitly coordinated with lesion-associated signals or stage-specific therapeutic requirements. Adaptability emerges when multiple responsive modules are integrated to recognize changes in lesion status and coordinate material degradation, cargo release, antibacterial activity, immune regulation, angiogenesis, and osteogenic support in a stage-matched manner. Accordingly, Sections 3.1–3.5 describe the major endogenous and exogenous responsive building blocks, whereas Section 3.6 focuses on multi-responsive and sequential-release systems that more closely represent adaptive hydrogel platforms. Endogenous pathological cues provide an important basis for responsive hydrogel design, but their presence should not be equated with reliable material activation. Local pH, ROS levels, enzyme activities, and oxygen tension in infected bone defects are spatially heterogeneous and change with bacterial burden, vascular perfusion, tissue necrosis, host immune responses, disease stage, and previous treatment. A cue that is biologically detectable may therefore be too weak, transient, or localized to exceed the activation threshold of a hydrogel throughout the defect. In addition, trigger conditions used *in vitro* are often simplified approximations and may not represent the full range encountered *in vivo*. Responsive systems should consequently be evaluated over physiologically plausible trigger ranges, with clear reporting of activation thresholds, response kinetics, dynamic ranges, and off-state leakage under non-infected conditions.

### 3.1 pH-responsive hydrogels

The local microenvironment of infected bone defects is frequently acidic, which is closely associated with the accumulation of bacterial metabolites, enhanced glycolysis of inflammatory cells, tissue necrosis, and insufficient local blood supply. Acidic conditions not only influence antimicrobial activity and cellular metabolic states but also interfere with bone matrix mineralization and osteogenic repair (Shen et al., 2026). Therefore, pH-responsive design represents one of the most widely used strategies in hydrogel-based therapies for infected bone defects. pH-responsive hydrogels are commonly constructed by introducing acid-labile chemical bonds, protonatable groups, or pH-sensitive nanocarriers. Under acidic conditions within infected lesions, these hydrogels may undergo network relaxation, accelerated degradation, or disassembly of drug-loaded particles, thereby promoting the localized release of antibacterial agents, metal ions, or immunomodulatory factors. At the chemical bond level, commonly used acid-sensitive dynamic covalent bonds include imine bonds (C = N), hydrazone bonds, and boronate ester bonds, whose hydrolytic stability increases in this order. Imine bonds can be rapidly cleaved under mildly acidic conditions and are therefore suitable for early rapid release. Hydrazone bonds exhibit moderate stability and are often used for sustained-release systems. Boronate ester bonds, formed through reversible condensation between boronic acids and cis-diols, can dynamically dissociate under acidic or high-ROS conditions, making them attractive chemical motifs for dual pH/ROS-responsive hydrogel design (Condò et al., 2024; Terriac et al., 2024). A chitosan-based composite device using PDMAEMA as a pH-sensitive driving element has been shown to undergo pronounced volumetric swelling under infection-mimicking acidic conditions of pH 5–6, thereby triggering on-demand minocycline release. This system effectively inhibited *S. aureus* while exhibiting no obvious cytotoxicity toward osteoblasts under infection-mimicking experimental conditions. It therefore provides proof-of-concept support for pH-triggered antimicrobial release, but does not constitute direct validation in an infected bone defect model (Chen H. et al., 2023). During the early stage of infection, pH-triggered release can increase the local concentration of antibacterial components within the lesion and enhance the killing of both planktonic bacteria and biofilm-associated bacteria. Beyond antibacterial drug delivery, pH-responsive design can also be integrated with immune regulation and osteogenic repair. Certain metal ions or nanomaterials released under acidic conditions can exert antibacterial, anti-inflammatory, and pro-osteogenic effects. For example, Cu^2+^ exhibits broad-spectrum antibacterial activity by disrupting bacterial cell membranes, interfering with bacterial DNA double-helix structures, and inducing intracellular ROS production (Shen et al., 2021). A pH-responsive silk fibroin coating suppressed Cu^2+^ release under physiological conditions and accelerated release under acidic conditions (Zhang et al., 2025b). As a surface-coating system rather than an injectable or defect-filling hydrogel, this study provides a transferable design principle for acidity-regulated ion delivery rather than direct evidence for infected bone defect repair. Mg^2+^ can exert direct anti-inflammatory effects by inhibiting NF-κB nuclear translocation and phosphorylation and downregulating pro-inflammatory cytokines such as IL-1β and IL-6. It can also promote macrophage transition from an M1-like to an M2-like phenotype and indirectly drive mesenchymal stem cell osteogenic differentiation and mineralization through macrophage-derived IL-8 (Gu et al., 2026). Zn^2+^ can regulate macrophage polarization toward an anti-inflammatory phenotype, induce the secretion of pro-osteogenic cytokines, and enhance BMSC osteogenic differentiation and osseointegration. Zn^2+^-containing biodegradable alloys have increased M2-associated macrophage markers and promoted early vascularized bone formation in non-hydrogel implantation models (Yi et al., 2025). These findings support the immunomodulatory potential of Zn^2+^ release but do not establish efficacy in infected bone defects. Sr^2+^ also possesses multiple functions, including promoting M2-like macrophage polarization, improving the inflammatory bone microenvironment, and simultaneously enhancing angiogenesis and osteogenesis (Li et al., 2025b). It should be noted that pH-responsive systems should be designed to avoid excessive burst release and cytotoxicity caused by high local concentrations. In addition, the response threshold of the material should be carefully matched to the actual pH fluctuation range within infected lesions. Local pH may vary within the same lesion and may partially normalize after debridement or antimicrobial treatment; therefore, mildly acidic conditions used *in vitro* should not be regarded as universal trigger values for infected bone defects. Evidence from metal alloys, inorganic biomaterials, and noninfected implantation models is included here only to illustrate the osteoimmunomodulatory actions of released ions and should not be interpreted as direct hydrogel evidence in infected bone defects.

### 3.2 ROS-responsive and antioxidant hydrogels

Infected bone defects are commonly associated with pronounced oxidative stress. Neutrophils and macrophages generate large amounts of ROS during antibacterial responses to eliminate invading pathogens (Roy et al., 2025). However, prolonged and excessive ROS accumulation can induce lipid peroxidation, mitochondrial damage, protein oxidation, and DNA damage, thereby inhibiting the proliferation and osteogenic differentiation of osteoblasts and bone marrow mesenchymal stem cells (BMSCs) (Huang Q. et al., 2025). Excessive ROS can also amplify inflammatory responses and maintain macrophages in a prolonged pro-inflammatory state, further aggravating bone resorption and tissue destruction (Bai et al., 2025). Based on these features, ROS-responsive hydrogels can participate in infected bone defect repair through two major strategies. The first strategy involves ROS-triggered release systems. In these hydrogels, ROS-sensitive chemical bonds are incorporated into the crosslinked network or drug-conjugated structures. Under high-ROS conditions, these bonds undergo cleavage or degradation, enabling the selective release of antibacterial drugs, anti-inflammatory factors, or osteoactive components. Common ROS-sensitive structures include thioethers, selenoethers, boronate esters, and thioketals (Pan et al., 2026). In a noninfectious inflammatory joint model, methylprednisolone-loaded ROS-responsive nanoparticles reduced pro-inflammatory cytokine release through NF-κB regulation and attenuated joint destruction, providing mechanistic support for inflammation-responsive delivery rather than direct evidence in infected bone defects (Lu et al., 2023). The second strategy involves ROS-scavenging hydrogels. In this approach, the hydrogel itself or its loaded components possess antioxidant capacity and can directly reduce local ROS levels, thereby mitigating oxidative stress-induced injury. Sun et al. developed a multifunctional curcumin-loaded nanocoating (Zn-Cur-TN) that scavenged intracellular ROS and exhibited antibacterial, anti-inflammatory, angiogenic, and osteogenic effects (Sun et al., 2025). Because this system was evaluated as a surface nanocoating rather than as a defect-filling hydrogel platform, it is cited here as supportive material evidence for combining redox regulation with antibacterial and regenerative functions. ROS-scavenging hydrogels have also improved stem-cell activity and osteointegration in an osteoporosis model (Ding et al., 2023). These results support their regenerative and immunomodulatory potential under compromised bone conditions, but antibacterial activity, biofilm control, and infected bone regeneration were not evaluated in that model. In infected bone defects, ROS-responsive and antioxidant regulation has dual significance. On the one hand, preserving an appropriate level of ROS-mediated antibacterial activity during the early stage of infection is beneficial for pathogen clearance. On the other hand, timely scavenging of excessive ROS can reduce host tissue damage and facilitate the transition from inflammation toward tissue repair. Notably, low levels of ROS in the later stage of infection may themselves act as signaling molecules that promote M2-like macrophage polarization and initiate tissue repair. Therefore, excessive or sustained ROS elimination is not always beneficial. Material design should take into account the intensity and duration of ROS regulation required at different disease stages (Wang et al., 2026a). Accordingly, ideal ROS-responsive hydrogels should not simply aim for complete antioxidation. Instead, they should regulate the magnitude and duration of oxidative stress in a stage-adapted manner. By reducing excessive ROS levels, these hydrogels can promote the transition of macrophages from a persistent pro-inflammatory state toward repair-associated phenotypes, while improving the survival and differentiation microenvironment of osteoblasts and BMSCs. Although oxidative stress is associated with bone infection, the local concentrations and spatial distribution of individual ROS species remain insufficiently defined, and commonly used *in vitro* H_2_O_2_ concentrations require validation against actual *in vivo* trigger ranges.

### 3.3 Enzyme-responsive hydrogels

Multiple enzymes are abnormally expressed in the local microenvironment of infected bone defects, including bacteria-derived proteases, hyaluronidases, and collagenases, as well as matrix metalloproteinases (MMPs) secreted by host inflammatory cells and osteogenesis-related cells. These enzymes participate in bacterial tissue invasion, biofilm remodeling, extracellular matrix degradation, and inflammatory tissue destruction (Suresh et al., 2019). At the same time, they provide lesion-specific triggering signals for the design of enzyme-responsive hydrogels. Compared with pH- or ROS-responsive systems, the major advantage of enzyme-responsive hydrogels lies in their high biological specificity. Enzymatic reactions exhibit strict substrate selectivity, enabling materials to be designed to respond only in lesion regions where specific enzymatic activity is elevated. This feature allows more precise and spatially confined drug release (Wang et al., 2026b). In general, enzyme-responsive hydrogels should meet two basic design requirements. First, substrates with high affinity for the target enzyme must be incorporated into the hydrogel network. Second, the enzyme–substrate reaction should induce substantial changes in hydrogel properties, such as degradation or swelling, thereby enabling effective cargo release. Typically, enzyme-responsive hydrogels are constructed by introducing peptide sequences, natural polysaccharides, or protein-based structures that can be degraded by specific enzymes, allowing local hydrogel degradation and release of antibacterial or repair-related components in infected or inflamed tissues (Patel et al., 2024). Hyaluronidase (HAase), for example, is a characteristic virulence-associated enzyme overproduced by *S. aureus* during infection and can serve as an infection-triggered signal. HAase-responsive hydrogels constructed using hyaluronic acid as the backbone can undergo on-demand degradation and drug release in response to bacteria-secreted HAase, while remaining stable under non-infected conditions. This enables precise “infection-triggered and smart drug delivery” control (Liu M. et al., 2024). In an animal model of infected bone defects, a honeycomb-inspired HAase-responsive hyaluronic acid hydrogel/vancomycin composite scaffold was shown to effectively inhibit MRSA infection within 14 days in a rabbit tibial defect infection model while promoting BMSC osteogenic differentiation, thereby achieving synergistic anti-infective and bone-repair effects (Fu J. et al., 2025). MMP-responsive systems have been explored in noninfectious osteoarthritis models, whereas microenvironment-responsive antibacterial platforms have been evaluated in periodontitis (Liu Y. et al., 2026; Xu Y. et al., 2023). These studies demonstrate transferable principles for enzyme-sensitive degradation and localized release, but their efficacy against long-bone infection, mature biofilms, and infected bone defects requires direct validation. In a noninfected bone-regeneration model, an MMP-cleavable peptide-crosslinked PEG hydrogel released phosphatidylserine and promoted repair-associated macrophage signaling and osteogenic differentiation, supporting the osteoimmunomodulatory potential of this design (Zhang et al., 2024). Because enzymatic profiles vary across infection stages, pathogen types, and tissue repair phases, enzyme-responsive hydrogels hold promise for achieving more precise spatial release and stage-specific therapy. However, this strategy also faces several challenges, including interindividual differences in lesion enzyme activity, possible cross-degradation among different enzymes, and difficulty in predicting *in vivo* response intensity (Tian et al., 2026). Therefore, enzyme-responsive hydrogels may be more suitable when combined with pH-, ROS-, or other stimulus-responsive mechanisms to construct multi-responsive platforms, thereby improving the accuracy and stability of local release in infected bone defects. Enzyme expression does not necessarily reflect the catalytic activity available for hydrogel degradation, because pathogen type, endogenous inhibitors, tissue diffusion, and disease stage may substantially influence the effective triggering intensity.

### 3.4 Hypoxia-responsive and oxygen-releasing hydrogels

Local hypoxia is commonly observed in infected bone defects and is closely associated with vascular destruction, tissue edema, increased oxygen consumption by inflammatory cells, sequestrum formation, and insufficient vascular reconstruction within the defect region (Wang et al., 2026c). Hypoxia not only affects bacterial metabolism and biofilm formation but also regulates immune cell function, angiogenesis, and bone regeneration. Moderate hypoxia can induce HIF-1α protein accumulation and upregulate the expression of angiogenic factors such as VEGF, thereby promoting early vascularization. However, prolonged and severe hypoxia suppresses osteoblast activity, weakens the osteogenic differentiation capacity of BMSCs, and delays bone matrix formation (Li J. et al., 2026). In response to the hypoxic microenvironment, hydrogel design mainly involves two strategies. The first strategy is hypoxia-responsive hydrogels, which rely on hypoxia-sensitive structures or carriers to enable selective drug release in low-oxygen lesions. Hypoxia- and MMP-responsive hydrogel microspheres, such as HAM-SA@HCQ, have demonstrated controlled drug delivery in a noninfectious osteoarthritis model (Zhou et al., 2024). This work provides a transferable dual-trigger design principle, but it did not evaluate bacterial infection, biofilms, structural bone defects, or bone-regeneration outcomes and therefore cannot be regarded as direct evidence for infected bone defect repair. The second strategy is oxygen-releasing hydrogels. These systems improve local oxygen supply by incorporating calcium peroxide, magnesium peroxide, catalase, hemoglobin-like oxygen carriers, perfluorocarbons, or other oxygen-generating/releasing components, thereby promoting cell survival, angiogenesis, and osteogenic repair (Sarkar et al., 2024; Peng et al., 2021). Oxygen-releasing strategies have considerable potential in infected bone defects. Improved oxygen supply not only enhances local cellular metabolism and osteogenic capacity but also promotes the recovery of vascular endothelial cell function and supports the formation of new vascular networks. Yang et al. (2024) developed a 3D-printed bioactive hydrogel scaffold by integrating oxygen-generating nanoparticles into a hybrid double-network hydrogel structure. This scaffold encapsulated CaO_2_ nanoparticles and achieved stable and sustained oxygen release. With the assistance of Zn^2+^, it further promoted angiogenesis and osteogenic differentiation, thereby accelerating bone regeneration throughout the defect region (Yang et al., 2024). Changes in oxygen availability also influence macrophage immunometabolism and inflammatory status. At the molecular level, hypoxia stabilizes HIF-1α and activates mTORC1 signaling, thereby upregulating glycolytic enzymes and reprogramming macrophage metabolism toward an M1-like metabolic profile dominated by glycolysis and the pentose phosphate pathway. Persistent hypoxia therefore reinforces the pro-inflammatory macrophage phenotype at the metabolic level (Li et al., 2025a; Wang et al., 2017). In contrast, M2-like macrophages mainly depend on oxidative phosphorylation and fatty acid oxidation for energy production, and the maintenance of this reparative phenotype requires adequate oxygen availability. Moderate improvement of local oxygen supply may restore aerobic metabolism, promote the metabolic transition from glycolysis toward oxidative phosphorylation, facilitate macrophage polarization from an M1-like to an M2-like state, and accelerate inflammation resolution and tissue repair. In an animal model of osteomyelitis, SETD2 was shown to regulate glycolysis in infection-associated macrophages by modulating HIF-1α expression, further supporting the central role of the oxygen-sensing–metabolic axis in immune regulation during bone infection (Zhu et al., 2024). Although hypoxia has relatively strong biological support in infected bone, oxygen tension remains spatially heterogeneous and may change after debridement, revascularization, or oxygen-releasing treatment, requiring careful matching between lesion oxygen levels and material activation thresholds.

### 3.5 Externally triggered photothermal and photodynamic hydrogels

Unlike hydrogels activated by endogenous pathological cues such as acidic pH, elevated ROS, abnormal enzyme activity, or hypoxia, photothermal and photodynamic hydrogels primarily depend on externally applied light stimulation. These systems therefore represent externally triggered rather than strictly microenvironment-responsive therapeutic strategies. Nevertheless, their externally controllable activation enables precise temporal regulation and can be integrated with endogenous microenvironment-responsive mechanisms to construct adaptive hydrogel platforms for infected bone defect repair. After the incorporation of polydopamine, black phosphorus, MXenes, gold nanomaterials, carbon-based materials, porphyrin-based photosensitizers, or other photoactive components, hydrogels can generate localized thermal effects or reactive oxygen species under near-infrared irradiation, thereby killing bacteria, disrupting biofilms, and improving antibacterial efficacy (Hu et al., 2024; Xiao et al., 2025a). Photothermal therapy (PTT) exerts multilayered biological effects by increasing the local temperature, and different temperature windows correspond to different therapeutic purposes. Temperatures of 45 °C–50 °C can damage bacterial cell membranes and induce protein denaturation, resulting in strong bactericidal effects against pathogens such as *S. aureus* and *E. coli*. In contrast, mild hyperthermia at 40 °C–45 °C can promote cell viability, improve local blood flow, and support bone tissue repair. However, temperatures above 60 °C may cause coagulative necrosis of host tissues (Xiao et al., 2025a; Chen X. et al., 2025). Based on this principle, multistage photothermal treatment strategies that precisely switch between different temperature windows by controlling irradiation intensity and duration have been validated in CS/HC/HA/BP composite hydrogels. In this system, 49 °C was used for targeted antibacterial treatment, whereas 42 °C was applied to promote bone regeneration, achieving stage-specific integration of antibacterial activity and osteogenic promotion within a single material platform (Zhao Y. et al., 2023). In conventional PTT, bacteria may acquire thermal tolerance by activating heat shock proteins (HSPs), which reduces the bactericidal efficacy of moderate-temperature PTT. To address this issue, a BP@MnO_2_ nanocomposite system used Mn-based nanozyme activity to generate ROS and reduce intracellular ATP levels in bacteria, thereby preventing sufficient HSP synthesis to resist thermal stress. This strategy enabled efficient killing of multidrug-resistant bacteria under mild photothermal conditions while maintaining tissue safety (Wang F. et al., 2023). In addition, conventional PTT commonly uses NIR-I lasers in the 750–900 nm range, which have limited penetration depth in bone and soft tissues. The development of NIR-II window photothermal agents, such as the Pt@V_2_C MXene nanoplatform developed by He and colleagues, enables deeper tissue photothermal activation and provides an improved direction for treating deep bone infections (He et al., 2024). In terms of integrating immunomodulatory functions, black phosphorus–polydopamine (BPPD) composite hydrogels have been shown in both *in vitro* and *in vivo* experiments to effectively kill bacteria under mild NIR irradiation. Beyond antibacterial activity, these hydrogels induced macrophage polarization toward an M2-like reparative phenotype, promoted the expression of angiogenic and osteogenic factors, and recruited endogenous stem cells to the defect site, thereby achieving multifunctional synergy among photothermal antibacterial therapy, immune regulation, and tissue repair (Wu M. et al., 2024). Du and colleagues constructed a degradable hydrogel through photopolymerization. This PBCB hydrogel was loaded with berberine (BR)-containing mesoporous polydopamine nanoparticles (MPDA) and in situ-formed Cu and Bi nanoparticles. The resulting system not only inhibited biofilm formation and promoted biofilm disruption but also reduced virulence factor expression and promoted infected wound healing (Xiao et al., 2025b). Although this study supports its antibacterial and antibiofilm activity, it did not reproduce deep bone infection, necrotic bone, load-bearing requirements, or structural bone-regeneration outcomes. Despite their strong antibacterial performance under controlled experimental conditions, the translational readiness of PTT and PDT for infected bone defects remains limited. Most reported bactericidal rates are obtained *in vitro* under direct and uniformly distributed irradiation, conditions that do not adequately reproduce the optical and anatomical barriers encountered in clinical bone infection. In deep lesions, incident light may be absorbed, scattered, or attenuated by skin, muscle, scar tissue, cortical bone, necrotic tissue, and inflammatory exudates before reaching the target material. Metallic internal fixation and irregular postoperative cavities may further complicate the irradiation path and produce spatially heterogeneous energy deposition (Wang et al., 2026d). Heat-dose control is another major concern. The antibacterial effect of PTT depends on the temperature achieved and the duration of exposure, but temperature distribution within irregular infected defects may be highly nonuniform. Insufficient heating may fail to eradicate bacteria and may promote bacterial thermotolerance, whereas excessive heating may damage osteoblast-lineage cells, vascular endothelial cells, nerves, and surrounding soft tissues. Surface temperature measurements alone may not accurately reflect the temperature within deep bone or at the material–tissue interface. Therefore, spatially resolved thermal monitoring and standardized reporting of irradiation wavelength, power density, exposure duration, distance, and tissue temperature are required. Therefore, the synergistic combination of PTT and PDT represents an important strategy to overcome the limitations of each modality. Photothermal effects can increase bacterial membrane permeability and promote photosensitizer penetration into deeper biofilm layers, thereby enhancing ROS-mediated bacterial killing by PDT. Meanwhile, ROS generated by PDT can synergize with thermal effects to disrupt bacterial defense systems. Dual-functional PTT/PDT systems, such as carbon nanotube–zinc phthalocyanine platforms, have achieved bactericidal rates exceeding 97% in drug-resistant bacterial biofilm models (Wu G. et al., 2024; Huang Y. et al., 2024). PDT faces additional limitations because its efficacy depends on adequate oxygen availability, photosensitizer localization, and light penetration. These requirements may be difficult to satisfy in hypoxic, poorly perfused, and necrotic infected bone tissue. Moreover, the feasibility and safety of repeated irradiation remain uncertain, particularly when lesions are covered by thick soft tissues or require stable internal fixation. NIR-II and implantable or fiber-assisted light-delivery strategies may improve access to deeper lesions, but current evidence remains predominantly preclinical. Accordingly, PTT and PDT should presently be regarded as locally controllable adjunctive strategies for anatomically accessible lesions or surgically exposed defect cavities, rather than as broadly applicable alternatives to debridement, antimicrobial therapy, stable fixation, and bone reconstruction.

### 3.6 Multi-responsive and sequential-release hydrogels

The pathological microenvironment of infected bone defects is not driven by a single factor, but rather by the combined presence of acidic pH, elevated ROS, hypoxia, abnormal enzymatic activity, biofilms, and sustained release of inflammatory cytokines. Therefore, single-responsive hydrogels are often insufficient to fully meet the complex demands of infection-associated bone repair. By integrating pH-, ROS-, enzyme-, hypoxia-, temperature-, or photothermal-responsive mechanisms, multi-responsive hydrogels can achieve more precise cargo release and functional regulation in response to different pathological cues or external stimuli. Multifunctional hydrogel systems that combine sequential drug release, ROS scavenging, photothermal activation, and cell delivery have shown higher bacterial clearance efficiency, accelerated bone healing, and reduced infection recurrence compared with conventional therapeutic approaches in preclinical models (Zhang et al., 2025a; Lyu et al., 2026). The core value of multi-responsive design lies not in enforcing a fixed three-stage sequence, but in rebalancing therapeutic functions according to changes in lesion status. Infection control, immune remodeling, vascular reconstruction, and osteogenic repair should be understood as overlapping functional domains. Their relative dominance may change in either direction, particularly in chronic osteomyelitis and implant-associated infection, where residual bacteria, biofilms, inflammatory injury, hypoxia, and attempted regeneration frequently coexist. Traditional strategies often overemphasize M2 macrophage-mediated bone regeneration in the later stage, while overlooking the indispensable role of M1-driven immune clearance in pathogen elimination during the early phase of infection. Therefore, truly effective sequentially regulated hydrogels should maintain an appropriate pro-inflammatory immune response during the early stage rather than immediately and completely suppressing inflammation (Jin et al., 2026). During the early phase, hydrogels should preferentially release antibiotics, antimicrobial peptides, metal ions, or photothermal antibacterial components to kill planktonic bacteria, disrupt biofilms, and promote pathogen clearance. A vancomycin-loaded and BMP-2 peptide-grafted bifunctional poly(amino acid) hydrogel has been validated in a rabbit radial infected bone defect model. In this system, rapid early vancomycin release effectively controlled infection, whereas subsequent degradation of the hydrogel backbone released BMP-2 signals to promote osteogenesis, thereby achieving coordinated infection control and bone reconstruction within a single therapeutic platform (Cui et al., 2026; Zha et al., 2024). During the intermediate phase, antioxidant components, immunomodulatory factors, or nanozymes can be released to alleviate excessive inflammation and oxidative stress and to promote the transition of macrophage functions from a sustained pro-inflammatory state toward a repair-associated state. A NIR/pH dual-responsive Cu^2+^–MXene system demonstrated antibacterial and pro-angiogenic effects in an infected wound context, providing supportive evidence for coupling externally triggered antibacterial activity with immune regulation (Li Q. et al., 2026). Separately, a ROS-responsive EGCG/BMP-2 hydrogel achieved sequential inflammatory regulation and osteogenic delivery in an osteoporotic bone-defect model (Xiao P. et al., 2025). These studies illustrate complementary design principles, but neither alone constitutes direct validation of full-stage repair in infected bone defects. PDGF and BMP-2 exhibit antagonistic temporal interactions; premature PDGF release may weaken the osteoinductive effect of BMP-2. This suggests that angiogenic factor release should precede osteogenic factor delivery, allowing vascularization to be established before osteogenic signaling is enhanced (Lv et al., 2023). PTT-assisted pulsatile drug release represents another engineering approach for sequential integration. By controlling high antibacterial doses followed by lower pro-osteogenic doses under mild photothermal stimulation, this strategy enables functional switching from infection control to bone regeneration (Wu et al., 2025). Taken together, multi-responsiveness and sequential release are key features that distinguish adaptive hydrogels from conventional local drug carriers. Their design should not merely focus on loading multiple components simultaneously, but should rationally arrange the release sequence, release intensity, and release duration of different functional modules according to the pathological requirements of different stages of infected bone defect repair. Recent studies have integrated real-time pH monitoring into bilayer hydrogel structures, enabling assessment of lesion repair status through outer-layer pH sensing and demonstrating the feasibility of theranostic platforms for infected wound management (Nagay et al., 2026; Li et al., 2024). For a hydrogel to move beyond preprogrammed sequential release toward genuine adaptability, functional switching should be linked to measurable changes in lesion status rather than to elapsed time alone. Candidate indicators of persistent infection include viable bacterial burden, bacterial metabolic or virulence products, biofilm-associated signals, lesion acidification, and bacteria-derived enzyme activity. A shift toward inflammation-resolving intervention may be considered when infection-related signals show a sustained decrease but excessive ROS and pro-inflammatory cytokines, such as TNF-α, IL-1β, and IL-6, remain elevated. Stronger angiogenic and osteogenic support may become appropriate when infection control is maintained, destructive inflammation decreases, oxygen tension or perfusion improves, and regenerative insufficiency becomes the dominant limitation. Nevertheless, no individual biomarker can reliably define these transitions. Acidic pH, ROS, matrix-degrading enzymes, and hypoxia may also occur during sterile inflammation, material degradation, or normal tissue remodeling. Multimodal sensing or logical gating based on combinations of bacterial, inflammatory, metabolic, and vascular signals may therefore be more reliable than a single-trigger threshold. Material degradation, swelling, fluorescence, or cargo-depletion states may assist temporal control, but they should not be regarded as surrogates for biological recovery unless they are correlated with bacterial burden, inflammatory status, perfusion, and bone-regeneration outcomes. At present, universally validated transition thresholds are lacking, and most reported sequential-release hydrogels remain preprogrammed rather than genuinely feedback-regulated (Figure 2; Table 1).

**FIGURE 2:**
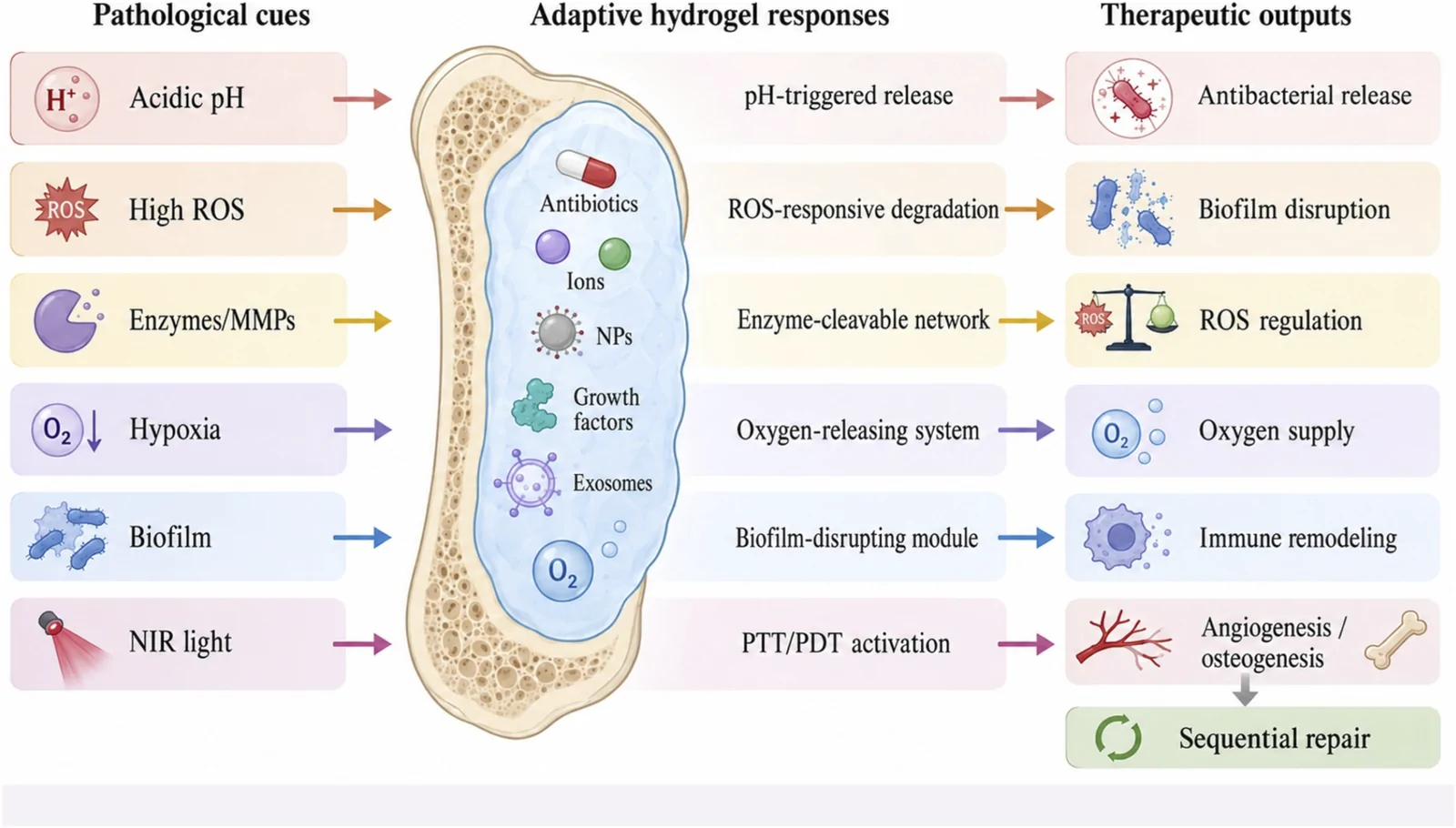
Responsive building blocks for adaptive hydrogel intervention in infected bone defects. Endogenous pathological cues, including acidic pH, elevated ROS, abnormal enzyme activity, hypoxia, and biofilm-associated signals, can trigger material degradation, cargo release, oxygen modulation, antioxidant activity, or antibiofilm effects. Exogenous stimuli, particularly near-infrared light, provide additional temporal control through photothermal or photodynamic activation. These stimuli-responsive mechanisms constitute functional modules that enable hydrogels to dynamically adapt to the pathological microenvironment through on-demand activation, sequential release, or multi-responsive coordination.

**TABLE 1:** Comparison of responsive strategies for infected bone defect repair.

Responsive strategy	Trigger category	Principal response	Best-fit phase	Main therapeutic value	Key limitation
pH-responsive	Endogenous	Acid-labile degradation or drug release	Early antibacterial phase	Localized antimicrobial delivery	Variable lesion pH and burst release
ROS-responsive/scavenging	Endogenous	ROS-triggered release or ROS removal	Intermediate immune remodeling	Reduces oxidative injury and persistent inflammation	Excessive scavenging may impair antibacterial defense
Enzyme-responsive	Endogenous	Enzyme-mediated degradation and cargo release	Early or intermediate phase	Relatively lesion-specific activation	Variable enzyme levels and off-target degradation
Hypoxia-responsive/oxygen-releasing	Endogenous	Hypoxia-triggered release or oxygen generation	Intermediate-to-late repair	Supports angiogenesis and osteogenesis	Difficult oxygen-dose control and limited antibacterial activity
Biofilm-targeted	Infection-associated	Biofilm disruption and enhanced antimicrobial penetration	Early antibacterial phase	Reduces residual bacteria and recurrence	Mature biofilms remain difficult to eradicate
PTT/PDT hydrogels	Exogenous light	Heat or ROS generation	Early antibacterial phase	Externally controlled bacterial killing	PTT/PDT are limited by poor deep-tissue penetration, heterogeneous energy distribution, and insufficient clinical validation.
Multi-responsive/sequential-release	Integrated endogenous/exogenous cues	Stage-dependent release and functional switching	Entire repair process	Coordinates antibacterial, immune, and regenerative functions	High manufacturing and regulatory complexity

Conventional sustained-release or multifunctional hydrogels are not considered adaptive solely because they provide prolonged release or incorporate multiple therapeutic components. Single-cue systems are classified as responsive modules, whereas multi-responsive, cue-dependent sequential-release, feedback-regulated, or explicitly stage-matched systems more closely represent adaptive hydrogel platforms. Light-activated PTT/PDT, systems are classified as externally triggered rather than microenvironment-responsive.

## 4 Macrophage-mediated osteoimmune regulation

### 4.1 Dual roles of macrophages in infected bone defects

Macrophages are among the most critical immune cells in the microenvironment of infected bone defects. They participate not only in pathogen recognition and infection clearance but also in inflammation resolution, angiogenesis, and bone tissue repair (Lu et al., 2025). During the early stage of infection, macrophages recognize pathogen-associated molecular patterns (PAMPs) and damage-associated molecular patterns (DAMPs) through pattern recognition receptors (PRRs), leading to their activation toward a pro-inflammatory, M1-like state. Innate immune recognition varies according to pathogen type. Because *S. aureus* and MRSA are the predominant pathogens in infected bone defects, TLR2-mediated recognition is particularly relevant. Staphylococcal lipoproteins activate TLR2–MyD88 signaling and subsequently induce NF-κB- and AP-1-dependent inflammatory responses. In contrast, TLR4 primarily recognizes lipopolysaccharide from Gram-negative bacteria and activates MyD88- and TRIF-dependent pathways. Cytosolic NOD1 and NOD2 recognize peptidoglycan-derived fragments and mainly signal through RIPK2; this process should not be equated with direct activation of the NLRP3 inflammasome. NLRP3 signaling generally involves two distinct steps: TLR- or cytokine receptor-mediated NF-κB activation first primes the inflammasome by increasing NLRP3 and pro-IL-1β expression, whereas subsequent signals, including pore-forming bacterial toxins, ion flux, or cellular stress, promote NLRP3–ASC–caspase-1 complex assembly and the maturation of IL-1β and IL-18 (Luo R. et al., 2025). After inflammasome activation and caspase-1-dependent cytokine maturation, released IL-1β may further amplify local inflammatory signaling. However, this downstream feedback should be distinguished from the initial NF-κB-dependent priming step (Lin et al., 2026). Activated M1-like macrophages are characterized by high expression of CD80, CD86, and MHC class II molecules. They release inflammatory mediators such as TNF-α, IL-1β, IL-6, IL-12, nitric oxide, and ROS, thereby strengthening local antibacterial responses and recruiting additional immune cells into the infected region (Gu and Pang, 2025; Yi et al., 2024). Therefore, moderate pro-inflammatory macrophage activation in the early stage is necessary for limiting bacterial dissemination and promoting pathogen clearance. However, inflammatory responses in infected bone defects often fail to terminate in a timely manner. Persistent bacterial colonization, biofilm-associated products, residual necrotic bone, and bacterial adhesion to implant surfaces can continuously stimulate pathogen- and damage-recognition pathways. Depending on the causative pathogen, these signals may involve TLR2-dominant recognition of staphylococcal components, TLR4-dependent recognition of Gram-negative lipopolysaccharide, cytosolic NOD1/NOD2–RIPK2 signaling, and separate inflammasome-activating stress signals. Repeated or unresolved activation of these pathways can sustain NF-κB-associated inflammatory gene expression and promote prolonged macrophage inflammatory activation. As a result, macrophages may remain in a prolonged pro-inflammatory state (Zhang J. et al., 2023; Tominari et al., 2024). Persistently elevated TNF-α, IL-1β, and IL-6 not only aggravate local tissue damage but also promote RANKL expression and osteoclast differentiation, leading to enhanced bone resorption (Li et al., 2025a). In addition, co-culture studies of macrophages and BMSCs have directly shown that M1-like macrophages significantly inhibit the osteogenic differentiation of BMSCs through the secretion of pro-inflammatory factors, thereby causing prolonged stagnation of bone repair (Zhang D. et al., 2023). Thus, the pro-inflammatory response of macrophages in infected bone defects is highly stage-dependent: it is beneficial for antibacterial defense in the early stage, but when sustained, it becomes an important driver of bone destruction and regenerative failure. As the bacterial burden decreases and inflammation gradually resolves, macrophages need to shift toward repair-associated functional states (Shi et al., 2025). Functionally, M2-like macrophages secrete a variety of reparative factors, including IL-10, TGF-β, VEGF, PDGF, and OSM. IL-10 can inhibit osteoclastogenic activity and promote new bone formation by regulating the RANKL/OPG balance (Galliera et al., 2024; Hu et al., 2023; Wang et al., 2026e). TGF-β enhances osteoblast differentiation and bone mineralization through BMP/Smad and Wnt/β-catenin pathways (Pramoda et al., 2026). In terms of angiogenesis, M2-like macrophages promote endothelial cell proliferation, migration, and tube formation through paracrine secretion of VEGF, bFGF, placental growth factor, and angiopoietins (Li J. et al., 2026; Huang X. et al., 2024). In particular, high levels of PDGF-BB expressed by M2-like macrophages can recruit pericytes and mesenchymal stem cells, initiate vascular sprouting, and stabilize newly formed vessels, thereby achieving structural coupling between angiogenesis and osteogenesis (Guo et al., 2020). Furthermore, M2 macrophage-derived exosomal miR-21 can activate the PI3K/Akt–mTOR–HIF-1α–VEGF signaling axis in endothelial cells, promoting endothelial migration and tube formation and further supporting neovascularization. This contact-independent paracrine pathway of M2-mediated vascularization provides osteoblasts and BMSCs with a more favorable regenerative microenvironment (Huang S. et al., 2025). Taken together, macrophages are not merely inflammatory effector cells. Rather, they function as regulatory hubs throughout infection control, immune remodeling, vascular reconstruction, and bone regeneration in infected bone defects.

### 4.2 Beyond M1/M2: dynamic immune regulation during infection-associated bone repair

Traditionally, macrophages have often been categorized into pro-inflammatory M1 and anti-inflammatory/reparative M2 phenotypes. M1-like macrophages typically express markers such as iNOS, CD86, CD80, and CCR7 and secrete pro-inflammatory cytokines, whereas M2-like macrophages commonly express markers such as CD206, ARG1, and CD163 and release anti-inflammatory and repair-associated factors (Li et al., 2025a; Toita et al., 2022; Orecchioni et al., 2019). However, with the development of transcriptomics and single-cell sequencing technologies, the limitations of the M1/M2 dichotomy have become increasingly evident. Large-scale transcriptomic analysis of 299 human macrophage samples under different stimulation conditions revealed a continuous spectrum of activation states rather than two clearly separated poles (Xue et al., 2014). Xue et al. further performed transcriptomic network analysis of human monocyte-derived macrophages exposed to 28 distinct stimuli and found that macrophage activation states did not conform to a simple M1/M2 binary pattern. Instead, they displayed a spectrum-like distribution, with 49 co-expression modules associated with different stimuli being identified. Subsequent single-cell transcriptomic and mass cytometry studies have also shown that some macrophages can simultaneously express traditional M1- and M2-associated markers, suggesting the presence of mixed or intermediate activation states with both pro-inflammatory and immunoregulatory features (Xue et al., 2014; Mohamad et al., 2021). Gene regulatory network models further support the concept that M1 and M2 represent only two extremes within a continuous range of macrophage polarization states, and that multiple hybrid phenotypes widely exist under both physiological and pathological conditions (Palma et al., 2018). Therefore, in infected bone defects, it is not fully appropriate to define the therapeutic goal simply as suppressing M1 polarization and promoting M2 polarization. Importantly, direct experimental evidence has demonstrated the potential risk of prematurely promoting M2-like conversion. In a *S. aureus* infection model, specific knockdown of M1-associated regulatory genes to promote macrophage transition toward an M2-like state reduced bacterial clearance and significantly increased pathogen burden (Lathram and Radka, 2025). This finding directly confirms the indispensable role of M1-like functions during the active phase of infection and highlights the real risk that excessive pursuit of M2 polarization may weaken antibacterial immunity. The immune requirements of infected bone defects are clearly stage-dependent. During the early phase, when the pathogen burden is high, the host requires a sufficiently strong antibacterial immune response to promote bacterial phagocytosis, bacterial killing, and biofilm disruption. Premature or excessive induction of an anti-inflammatory state may lead to residual infection or recurrence. Conversely, after infection has been initially controlled, persistent activation of pro-inflammatory macrophages can aggravate bone resorption and suppress osteogenesis (Zhang et al., 2025a; Gou et al., 2024). Therefore, ideal immunomodulation should dynamically regulate macrophage functional states according to the different stages of infection-associated bone repair. Throughout this review, “M1-like” and “M2-like” are used only as operational terms when adopted in the cited studies based on conventional marker analyses. They do not represent discrete or stable macrophage populations. In other contexts, we use functional descriptions such as pro-inflammatory, antibacterial, inflammation-resolving, and repair-associated states, and interpret macrophage changes as dynamic, context-dependent transitions rather than a simple M1-to-M2 conversion. In this context, adaptive hydrogels are advantageous because they can coordinate macrophage functional transitions through localized responsive release and stage-specific delivery. When bacterial burden and biofilm activity remain high, hydrogels should support antibacterial drug delivery, pathogen clearance, and the necessary bactericidal functions of macrophages. As infection-related signals decline but persistent inflammatory and oxidative injury remains, antioxidant or immunomodulatory cargos may be released to promote inflammation resolution and repair-associated macrophage functions. When infection control is sustained and vascular or osteogenic insufficiency becomes dominant, the platform may further support endothelial activation, osteogenic differentiation, and bone matrix reconstruction. These functions may overlap, and reactivation of infection may require renewed prioritization of antibacterial activity (Yuqiang et al., 2026; Zhang et al., 2020; Si et al., 2025). These findings support lesion-state-dependent regulation of macrophage functions rather than unidirectional promotion of an M2-like phenotype.

### 4.3 Material-based strategies for hydrogel-mediated regulation of macrophage function

Hydrogel-mediated regulation of macrophage function mainly relies on two complementary strategies: shaping the immune microenvironment through the intrinsic physicochemical properties of the material itself, and actively inducing macrophage functional remodeling through the delivery of antibacterial, antioxidant, or immunomodulatory agents. In infected bone defects, hydrogels should not only achieve local antibacterial effects but also promote inflammation resolution and bone regeneration after infection control. Therefore, their design should balance anti-infective and immunomodulatory requirements (Zhang et al., 2025a). At the level of material properties, hydrogel composition, surface charge, pore structure, stiffness, viscoelasticity, degradation rate, and functional groups can all influence macrophage adhesion, migration, morphological changes, and polarization states (Jackson et al., 2025; Solbu et al., 2022; Butenko et al., 2024; Wu et al., 2026). Among these parameters, stiffness is an important regulator of macrophage responses. Softer matrices or matrices with elasticity close to that of physiological tissues generally favor anti-inflammatory or repair-associated phenotypes, whereas stiffer matrices are more likely to induce pro-inflammatory responses. However, this relationship is influenced by the material system, cell source, and stiffness range and should not be interpreted as a simple linear rule. Studies using polyacrylamide, PEG, GelMA, and alginate-based hydrogels have shown that changes in stiffness can significantly affect macrophage inflammatory responses and polarization states (Chen et al., 2020; Coser et al., 2025).

Pore structure also exerts immunomodulatory effects. For example, in microporous annealed particle scaffolds, pore sizes of 40 μm and 70 μm induced different degrees of macrophage spreading and expression of M1/M2-associated markers, suggesting that pore size not only affects cell infiltration but also acts as a structural signal for regulating macrophage function (Liu et al., 2023). Surface chemistry and material composition represent additional regulatory dimensions. Natural polymers such as gelatin, collagen, hyaluronic acid, alginate, and chitosan possess favorable biocompatibility and can provide a relatively mild microenvironment for immune cells and osteogenesis-related cells (Zhao L. et al., 2023; Chelu et al., 2025). In contrast, synthetic or composite hydrogels allow more precise tuning of crosslinking density, degradation rate, and mechanical properties. For instance, hyaluronic acid–dopamine secondary-crosslinked hydrogels can simultaneously modulate mechanical properties, antioxidant capacity, and macrophage polarization by adjusting dopamine concentration. Low dopamine concentrations favor inflammation suppression and M2-like polarization, whereas high dopamine concentrations may exhibit pro-inflammatory tendencies during the later stage after implantation (Pei et al., 2024). Such hydrogels also possess favorable adhesiveness and self-healing properties, making them suitable for tissue repair applications (Wang Y. et al., 2024). The supporting studies include diabetic wound and noninfected bone-regeneration models and therefore primarily demonstrate material–macrophage interactions and regenerative potential, rather than direct efficacy in infected bone defects. In addition, the lateral size and oxidation/reduction degree of graphene oxide can influence macrophage responses. Reduced graphene oxide can decrease oxidative stress and pro-inflammatory cytokine secretion, whereas certain large-sized graphene oxide sheets may induce M1-like inflammatory responses, indicating that surface redox status and nanostructural parameters must be carefully optimized (Cicuéndez et al., 2021; Ma et al., 2015). Beyond intrinsic material properties, hydrogels can serve as local delivery platforms for immunomodulatory components. Antioxidant molecules, polyphenolic compounds, polydopamine, cerium-based nanoparticles, manganese-based nanomaterials, and other nanozyme systems can scavenge excessive ROS, alleviate oxidative stress and inflammatory injury, and thereby promote macrophage transition from a pro-inflammatory state toward a repair-associated state (Song et al., 2025; Gong et al., 2025; Zhang B. et al., 2023). Cytokine delivery is another classical strategy. IL-4-loaded hydrogels can promote M2-like macrophage polarization and enhance BMSC osteogenic differentiation through TGF-β1/Smad signaling, thereby coupling immunomodulation with bone regeneration (Shi et al., 2025; Zhang et al., 2020). However, in infected bone defects, the timing of anti-inflammatory cytokine release is particularly critical. Excessive early release of IL-4, IL-10, or TGF-β may compromise pathogen clearance, whereas release after bacterial burden has decreased is more conducive to inflammation resolution and tissue repair. Therefore, cytokine delivery should be integrated with antibacterial modules, pH/ROS/enzyme-responsive release mechanisms, or sequential delivery systems to avoid conflict between anti-inflammatory repair and infection control (Zhang et al., 2025a; Song et al., 2025). Exosomes, miRNAs, and natural bioactive compounds have also emerged as important cargos for immunomodulatory hydrogels (Qin B. et al., 2024; Hwang and Lee, 2024). BMSC-derived exosomes can remodel macrophage function through miRNA-mediated mechanisms. For example, miR-23a-3p promotes the transition from an M1-like to an M2-like state by targeting IRF1 and improves early inflammatory responses at the bone–tendon interface (Li et al., 2022a). In the context of osteomyelitis, BMSC exosome-derived miR-223 has been shown to inhibit macrophage pyroptosis and inflammatory injury by regulating LACC1, providing more direct evidence for exosome-based therapy in infected bone microenvironments (Zhou et al., 2025). LPS-preconditioned BMSC-derived exosomes can enhance M2-like polarization through the TSG-6/NF-κB/NLRP3 pathway and improve adaptability to inflammatory microenvironments. Such preconditioning strategies can amplify the anti-inflammatory potential of exosomes while preserving their pro-reparative capacity (Li et al., 2022b). In addition, curcumin-loaded MSC-derived exosomes encapsulated within bisphosphonate-modified GelMA hydrogel microspheres can be sustainably released to promote macrophage transition toward repair-associated phenotypes while synergistically enhancing osteogenesis and angiogenesis (Si et al., 2025). Metal ions are also important functional components in hydrogel-based osteoimmune regulation. However, part of the available evidence for ion-mediated osteoimmunomodulation is derived from metal alloys, surface coatings, hydroxyapatite materials, or other non-hydrogel systems. These studies are included to support the biological effects and dose dependence of released ions, rather than as direct validation of hydrogel therapy in infected bone defects. Ions such as Mg^2+^, Zn^2+^, Sr^2+^, and Cu^2+^ not only participate in osteogenesis and vascularization but also influence the osteoimmune microenvironment by regulating inflammatory cytokine release, oxidative stress, and macrophage functional states (Luo Y. et al., 2025; Qin S. et al., 2024). Protein-crosslinked hydrogels with sustained Mg^2+^/Zn^2+^ release improved the osteoimmune microenvironment and bone regeneration in a noninfected bone-repair model (Chen et al., 2021). The findings support the hydrogel-based delivery of immunomodulatory ions, although antibacterial and antibiofilm efficacy remain to be validated in infected bone defects. Mg^2+^ can modulate macrophage function through TRPM7 channels, whereas Zn^2+^ exhibits mild anti-inflammatory and antibacterial properties (Chen Z. et al., 2025). Zn^2+^/Sr^2+^ co-assembled hydroxyapatite promoted BMSC osteogenic differentiation and calvarial defect repair in a noninfected bone-regeneration model (Zhong et al., 2022). This study provides supportive evidence for ion-mediated osteoimmune regulation rather than direct infected-bone-defect evidence. However, the immunological effects of metal ions are strongly concentration-dependent, and excessive Cu^2+^, Zn^2+^, or other metal-ion release may cause cytotoxicity or pro-inflammatory responses (Huo et al., 2024; Liu S. et al., 2024). Therefore, the key to designing metal ion-loaded hydrogels is not to maximize ion loading, but to achieve mild, sustained, and controllable release. This allows metal ions to exert antibacterial, osteogenic, and pro-angiogenic effects while avoiding aggravation of local inflammatory injury.

### 4.4 Macrophage regulation from the perspective of immunometabolism

The functional state of macrophages is closely linked to their metabolic programs. At the metabolic phenotype level, pro-inflammatory M1-like macrophages preferentially rely on glycolysis even under oxygen-sufficient conditions. This metabolic mode rapidly generates energy, NADPH, and biosynthetic precursors to support pro-inflammatory cytokine production and antibacterial effector functions. At the level of the tricarboxylic acid (TCA) cycle, M1-like macrophages exhibit two characteristic metabolic breaks. The first occurs at the level of isocitrate dehydrogenase (IDH), leading to citrate accumulation and itaconate production. The second occurs at the level of succinate dehydrogenase (SDH), resulting in succinate accumulation. Succinate further stabilizes HIF-1α, enhances glycolytic activation, and upregulates IL-1β production, while SDH also drives mitochondrial ROS generation. Together, these processes form a mutually reinforcing “glycolysis–HIF-1α–IL-1β–ROS” inflammatory circuit (Wculek et al., 2022; Viola et al., 2019; Pålsson-McDermott and O’Neill, 2025). In contrast, repair-associated M2-like macrophages mainly depend on oxidative phosphorylation (OXPHOS) and fatty acid oxidation (FAO) to maintain anti-inflammatory and tissue-repair functions (Liu et al., 2021). Pharmacological inhibition of the key glycolytic enzyme hexokinase using 2-deoxy-D-glucose (2-DG) has been shown to suppress M1-like transition and reduce pro-inflammatory cytokine secretion, indicating that metabolic reprogramming is a directly actionable target for regulating macrophage functional states (Cai et al., 2021). The local microenvironment of infected bone defects is often characterized by hypoxia, elevated ROS, lactate accumulation, nutrient deprivation, and exposure to bacterial metabolites. These factors can collectively drive macrophage metabolism toward glycolysis and maintain a pro-inflammatory state. HIF-1α is a key node linking hypoxia, glycolysis, and inflammatory responses. Moderate HIF-1α activation during the early stage of infection helps enhance macrophage glycolysis and antibacterial capacity, whereas persistent activation may amplify inflammatory injury and delay tissue repair (Li J. et al., 2026; Qiu et al., 2023). Lactate, a major product of glycolysis, can promote M1-like polarization at high concentrations by activating HIF-1α, thereby forming a metabolic positive feedback loop of “hypoxia–enhanced glycolysis–lactate accumulation–HIF-1α stabilization–further glycolytic activation” (Liu L. et al., 2026; Ma H. et al., 2024). However, lactate should not be simply defined as a unidirectional M1-promoting factor. It can participate in HIF-1α-related metabolic adaptation under hypoxic inflammatory conditions, but it may also induce repair-associated gene expression through epigenetic mechanisms such as histone lactylation (Yuan et al., 2025). Thus, in the infected bone microenvironment, lactate should be understood as a stage-dependent and context-specific immunometabolic signal. Notably, the TCA cycle itself also contains endogenous anti-inflammatory negative regulatory mechanisms. Itaconate can attenuate succinate accumulation by inhibiting SDH activity and can suppress JAK1 phosphorylation through cysteine modification mechanisms, thereby negatively regulating both M1 inflammation and M2 activation. This represents a potential class of metabolic targets for immunomodulation (Runtsch et al., 2022). Mitochondria are central organizers of this metabolic plasticity. Beyond their role as energy-producing organelles, mitochondria function as signaling platforms that integrate Ca^2+^, ROS, and metabolite signals to regulate epigenetic modification, gene expression, and cytokine production. Impaired mitochondrial OXPHOS is one of the metabolic features of M1-like macrophages, and mitochondrial ROS can further amplify inflammatory responses by damaging the autophagy–lysosome system (Li et al., 2025a; Zhang G. et al., 2026). Studies in diabetic bone defects and other tissue-regeneration contexts suggest that hydrogels can regulate macrophage metabolism by modifying local oxygen supply, redox balance, and mitochondrial function (Fu X. et al., 2025; Jiang et al., 2024). These findings provide mechanistic support for immunometabolic design, but their effects on bacterial clearance, biofilms, and infected bone regeneration require direct assessment. ROS-responsive or antioxidant hydrogels can decrease oxidative stress, improve mitochondrial function, and reduce sustained release of pro-inflammatory cytokines. In addition, certain metal ions, nanozymes, and natural bioactive compounds may influence macrophage immunometabolism by regulating redox balance, mitochondrial metabolism, and inflammatory signaling (Song et al., 2025; Fu X. et al., 2025). Compared with conventional phenotype-based regulation, interpreting hydrogel effects from an immunometabolic perspective helps explain why these materials can simultaneously modulate inflammation, vascularization, and osteogenic repair. Iron metabolism in the infected microenvironment is another important aspect of macrophage functional regulation. Nutritional immunity is a key component of innate host defense. The host reduces systemic iron availability through the hepcidin–ferroportin (FPN) axis, while macrophages at infected sites downregulate transferrin receptor (TFR) expression and actively remove iron from phagosomes through Nramp1, thereby limiting iron availability for intracellular pathogens. Locally produced lactoferrin and lipocalin-2 further chelate free iron within infected lesions, achieving coordinated iron sequestration (Marchetti et al., 2020; Je and Ep, 2013). Pathogens generally require iron for growth and biofilm formation, whereas host macrophages can restrict bacterial iron utilization by regulating iron uptake, storage, and release (Pereira et al., 2019). Certain antibacterial materials, such as gallium-based systems, exert antibacterial effects by interfering with bacterial iron metabolism, while changes in iron homeostasis may also influence macrophage oxidative stress and inflammatory responses (Sun W. et al., 2023; Goss et al., 2018). Therefore, future hydrogel design should further consider the interactions among macrophage iron metabolism, redox status, and energy metabolism, with the goal of achieving more precise osteoimmune regulation.

### 4.5 Interactions of macrophages with osteogenesis, osteoclastogenesis, and vascularization

Macrophage-mediated osteoimmune regulation ultimately needs to serve bone regeneration and remodeling. In bone tissue, a specialized population of tissue-resident macrophages, known as endosteal/periosteal macrophages or OsteoMACs, has been identified. These cells are typically characterized by an F4/80^+^CD169^+^Mac-2^−^/low phenotype. OsteoMACs are located adjacent to osteoblasts and form canopy-like structures covering mature osteoblasts, directly supporting osteoblast-mediated bone formation and matrix mineralization during bone homeostasis (Batoon et al., 2019). Depletion of OsteoMACs both *in vitro* and *in vivo* significantly reduces the bone-forming capacity of osteoblasts, confirming the indispensable role of bone-resident macrophages in maintaining skeletal anabolism (Weivoda and Bradley, 2023). In infected bone defects, the normal function of OsteoMACs may be disturbed by persistent inflammatory signals, further impairing the local osteogenic microenvironment. Macrophages form complex interaction networks with osteoblasts, osteoclasts, BMSCs, and vascular endothelial cells through the secretion of cytokines, chemokines, and growth factors (Li et al., 2025a; Zhang et al., 2025b). Persistently pro-inflammatory macrophages release large amounts of inflammatory mediators, suppress the expression of osteogenesis-related genes, and reduce ALP activity, collagen synthesis, and mineralization capacity. At the same time, these inflammatory mediators can promote RANKL expression, enhance osteoclast differentiation and bone resorption, and shift the defect microenvironment toward increased osteoclastogenesis and impaired osteogenesis (Huang et al., 2017; Lu et al., 2017; Hu et al., 2022). However, M1-like macrophages may also exert positive pro-osteogenic effects during the early phase of bone repair. For example, M1-like macrophages can promote osteoblast-mediated bone formation by secreting oncostatin M (OSM). In addition, the early pro-inflammatory microenvironment induced by M1-like activation helps recruit MSCs, osteoprogenitor cells, and vascular progenitor cells to fracture or defect sites and induces the early expression of BMP-2, RUNX2, and ALP. Thus, during the acute inflammatory phase of bone repair, M1-like macrophages may perform dual functions in debridement and repair initiation (Zhang et al., 2025a; Zhang D. et al., 2023). This suggests that early M1-like responses should not be blindly suppressed in stage-specific immunomodulatory strategies. As macrophages gradually shift toward repair-associated functional states, their effects on bone regeneration also change. Reparative macrophages can secrete IL-10, TGF-β, BMP-related factors, VEGF, and PDGF, thereby promoting BMSC migration, osteogenic differentiation, and matrix deposition (Muñoz et al., 2020; Kolliopoulos et al., 2025). For infected bone defects, this immune–bone metabolic coupling is particularly important. Only after infection is controlled, inflammation gradually resolves, and an osteogenesis-favorable microenvironment is restored can bone matrix reconstruction and mineralization proceed effectively. Macrophages are also closely involved in vascularization. Newly formed blood vessels provide oxygen, nutrients, and reparative cells to the bone defect region and are therefore essential for bone regeneration (Zhang D. et al., 2023; Gou et al., 2024). M2-like macrophages can secrete VEGF, PDGF, and other pro-angiogenic factors to promote endothelial cell migration, proliferation, and tube formation. In turn, angiogenesis improves local hypoxia and metabolic disorders, providing a more stable regenerative environment for osteoblasts and BMSCs. Therefore, macrophages not only regulate inflammatory status but also indirectly promote bone repair by supporting vascularization (Fan et al., 2023; Han et al., 2022; Shi et al., 2026). Hydrogels can enable continuous treatment from infection control to regenerative remodeling by regulating the interactions between macrophages and bone-related cells. Collectively, macrophages connect inflammatory regulation with osteoclast activity, osteoblast function, BMSC differentiation, and vascular reconstruction, thereby providing a biological basis for hydrogel-mediated osteoimmune regulation.

## 5 Antibacterial–immune–osteogenic coupling for regenerative remodeling

### 5.1 From monofunctional therapy to multi-process synergistic repair

Multifunctional hydrogels aim to combine antibacterial, immunomodulatory, vascular, and osteogenic functions within one local platform. Representative infected-defect studies have demonstrated the feasibility of temporal immunomodulation and integrated antibacterial–regenerative repair (Jin et al., 2026; Zhang et al., 2025c). However, adding multiple components does not necessarily produce additive benefit and may introduce functional redundancy, antagonistic biological effects, and unpredictable release kinetics. The design paradigm of new materials for bone infection repair has shifted from monofunctional antibacterial or osteogenic materials toward multifunctional integrated strategies that combine antibacterial efficacy, osteogenic promotion, and immunomodulatory capacity (Xiang et al., 2025). The advantage of adaptive hydrogel platforms lies in their ability to integrate multiple therapeutic modules within the same local microenvironment. Through material degradation, responsive release, and synergistic actions of functional components, different therapeutic processes can be coordinated in both time and space. In terms of release sequence, antibacterial modules should be actively released during the early phase to rapidly reduce bacterial burden, whereas immunomodulatory agents should be released simultaneously or with a slight delay. Premature suppression of inflammation may weaken M1-like macrophage-mediated pathogen clearance, whereas excessively delayed immunomodulation may allow the pro-inflammatory microenvironment to continuously damage osteoblasts and BMSCs (Zhang et al., 2025a). Sequentially immunomodulatory hydrogels have been shown to effectively remodel the osteoimmune microenvironment, suppress infection, and promote vascularized bone regeneration in a rat model of infected calvarial defects, demonstrating the synergistic therapeutic efficacy of temporal regulation strategies *in vivo* (Jin et al., 2026). In addition, biomimetic mineralized antibacterial hydrogels (imCOL1MA) loaded with BMSCs achieved rapid vascularized and innervated bone regeneration within only 4 weeks in a rabbit model of infected bone defects, supporting the preclinical feasibility of integrating antibacterial and osteogenic functions within a single platform. However, longer-term studies are required to determine the reproducibility, biosafety, and clinical relevance of this multicomponent strategy (Deng et al., 2025). Thus, hydrogels are no longer merely drug delivery vehicles. Despite these advantages, multifunctional integration should not be assumed to produce additive or synergistic benefits. The incorporation of multiple antibacterial, immunomodulatory, angiogenic, and osteogenic components may introduce functional redundancy or biological antagonism. For example, strong antibacterial ROS generation may conflict with the protection of osteogenic and vascular cells, whereas premature anti-inflammatory or antioxidant activity may weaken early pathogen clearance. Differences in the release kinetics, stability, and effective concentration ranges of individual components may also disrupt the intended therapeutic sequence. Therefore, multifunctional hydrogel systems should be evaluated not only according to the number of functions they provide, but also according to whether each component contributes a necessary, compatible, and temporally appropriate effect. Rather, they can function as local therapeutic platforms that participate in the entire regulatory process of infected bone defect repair.

### 5.2 Synergy between antibacterial therapy and immune regulation: from pathogen clearance to inflammation resolution

Antibacterial therapy is the foundation of infected bone defect repair, but the antibacterial process itself profoundly influences the local immune microenvironment. On the one hand, antibiotics, metal ions, antimicrobial peptides, nitric oxide (NO), and photothermal/photodynamic therapy can reduce bacterial burden and decrease the continuous stimulation of immune cells by bacterial virulence factors and biofilms, thereby helping to alleviate chronic inflammation (Zhang et al., 2025a). On the other hand, excessively strong or uncontrolled antibacterial stimulation may also cause host tissue injury. For example, high concentrations of metal ions, excessive ROS generation, or excessively high photothermal temperatures can damage osteoblasts, endothelial cells, and macrophages, ultimately delaying tissue repair (Song et al., 2025). M1-like macrophages themselves can generate large amounts of ROS and establish a pro-inflammatory positive feedback loop. After bacterial burden has decreased, if this ROS-driven inflammatory self-amplification mechanism is not effectively interrupted, inflammation may persist and continue to damage osteogenesis-related cells and vascular endothelial cells even after pathogens have been partially cleared (Li et al., 2025a; Song et al., 2025). Therefore, antibacterial modules should cooperate with immunomodulatory modules rather than function in isolation. In a burn wound model, an antibacterial and ROS-scavenging hydrogel promoted inflammation-resolving macrophage functions after infection control (Zhao et al., 2026). This finding supports the timing of antioxidant immunomodulation but does not directly establish bone-regenerative efficacy. Hydrogels can reduce host tissue injury associated with antibacterial therapy through localized and controllable release. A relatively high release dose during the early phase helps rapidly control infection, whereas stable low-dose release during the later phase can suppress residual bacterial proliferation and reduce infection recurrence. A representative example is a Ca^2+^/Zn^2+^ dual-ion sequential-release hydrogel. In this system, Early Ca^2+^ release supports antibacterial inflammatory activation, whereas subsequent sustained Zn^2+^ release favors inflammation-resolving and repair-associated macrophage functions, accompanied by enhanced osteogenic differentiation. In a rat model of infected calvarial defects, this system effectively remodeled the osteoimmune microenvironment and promoted vascularized bone regeneration, demonstrating a stage-specific antibacterial–immunomodulatory strategy based on the intrinsic plasticity of immune cells (Jin et al., 2026). A ROS-responsive co-delivery system achieved antibacterial and anti-inflammatory effects in a periodontitis model (Zhu et al., 2025). Because periodontitis involves infection-associated bone loss but differs from long-bone infected defects in anatomy, microbial ecology, mechanical loading, and defect size, this study should be regarded as partially relevant evidence. For photothermal or photodynamic antibacterial systems, moderate thermal effects and ROS generation are beneficial for bacterial killing and biofilm disruption. However, they should be combined with antioxidant or tissue-protective strategies to avoid sustained damage to host cells. Related nanozyme and phosphorene strategies have demonstrated ROS regulation, antibacterial activity, and tissue repair in general bone-regeneration or infected soft-tissue wound models (Song et al., 2025; Wang et al., 2026f; Dai et al., 2024). Direct validation in deep infected bone defects remains necessary. The synergy between antibacterial therapy and immune regulation is also reflected in the regulation of macrophage function. During the early stage of infection, appropriate pro-inflammatory activation supports macrophage phagocytosis and bacterial killing. After bacterial burden decreases, however, the persistent pro-inflammatory state must be terminated in a timely manner. Adaptive hydrogels can promote the transition of macrophages from sustained inflammatory activation toward repair-associated functional states by reducing bacterial stimulation, scavenging excessive ROS through systems such as Mn_3_O_4_ nanozymes to disrupt the ROS–M1 positive feedback loop, releasing anti-inflammatory factors such as IL-4, or delivering exosomes (Hwang and Lee, 2024; Li et al., 2025c; Pang et al., 2025). These findings indicate that antibacterial efficacy should be evaluated together with host-cell protection and the resolution of persistent inflammatory injury.

### 5.3 Immune–vascular–osteogenic coupling: from inflammation resolution to bone matrix reconstruction

The immune microenvironment is a key determinant of whether infected bone defects can progress into the regenerative phase (Yang and Liu, 2021). Under persistent inflammatory conditions, elevated pro-inflammatory cytokines suppress the expression of osteogenesis-related molecules and reduce the osteogenic differentiation capacity of BMSCs and osteoblasts. These inflammatory mediators can also promote RANKL expression, enhance osteoclastogenesis and bone resorption, and shift the defect microenvironment toward increased bone resorption and impaired bone formation (Shi et al., 2025; Xu J. et al., 2023). Therefore, delivery of osteogenic factors alone may not necessarily achieve satisfactory bone regeneration; rather, the local inflammatory immune microenvironment must be simultaneously improved. Macrophages serve as an important bridge between immune regulation and osteogenic repair, and their functional transition directly affects infection clearance, inflammation resolution, angiogenesis, and bone reconstruction (Gou et al., 2024). M1-like and M2-like macrophages should be regarded as immune regulatory units with stage-specific functions during infection-associated bone repair. Macrophage-derived exosomes are also important mediators of immune–osteogenic communication. Studies have shown that M2 macrophage-derived exosomes can promote BMSC osteogenic differentiation and inhibit adipogenic differentiation, possibly through the transfer of miRNAs, lncRNAs, proteins, and multiple reparative signaling molecules (Li et al., 2021; Wang D. et al., 2023). In animal models of bone defects, M2 macrophage-derived exosomes have shown potential to promote bone regeneration and vascularization. However, their cargo composition and effector pathways are model-dependent, and exosomes derived from macrophages with different polarization states may regulate BMSC migration, proliferation, and osteogenic differentiation through diverse molecular cargos (Wang D. et al., 2023). Hydrogel materials are not only carriers for antibacterial drugs or osteogenic factors, but can also function as local platforms for immune microenvironment regulation (Cao Z. et al., 2024). Through different design strategies, hydrogels can enhance antibacterial activity and pathogen clearance during the early stage of infection, and promote macrophage transition toward anti-inflammatory and reparative phenotypes during the middle and late stages, thereby providing a more favorable microenvironment for vascularization and osteogenic differentiation (Wu M. et al., 2024; Chen W. et al., 2023). For example, Yang et al. developed an inflammation microenvironment-regulating conductive hydrogel. This system used copper nanoparticles to achieve antibacterial activity and promoted the transition of macrophages from a pro-inflammatory M1-like phenotype toward a reparative M2-like phenotype, thereby improving the immune microenvironment of infected bone defects and further enhancing vascularization and bone regeneration (Yang et al., 2025). Vascularization is a critical link between immune regulation and bone regeneration. Infected bone defects are often accompanied by vascular destruction, tissue edema, and local hypoxia, and insufficient vascular network reconstruction restricts the entry of oxygen, nutrients, and reparative cells into the defect region (Li et al., 2025d; Grosso et al., 2017). Oxygen-releasing hydrogels are an important strategy for improving local hypoxia in infected bone defects (Shang et al., 2026). In noninfected calvarial defect models, oxygen-releasing hydrogels improved BMSC and endothelial-cell survival and enhanced vascularized bone formation (Xu et al., 2024). In a soft-tissue wound model, the oxygen-releasing QFCMC-OHA hydrogel combined antibacterial activity with accelerated tissue repair (Liu W. et al., 2026). Sequential SDF-1α/Mg^2+^ delivery and SDF-1α-loaded hydrogel–scaffold composites have also promoted cell recruitment, angiogenesis, and osteogenesis in noninfected bone-defect models (Li et al., 2022c; Wang C. et al., 2024). Collectively, these studies provide complementary evidence for oxygen delivery and vascular–osteogenic coupling, but their integrated efficacy against infection, biofilms, and structural bone loss requires validation in infected bone-defect models. Osteogenic modules ultimately translate antibacterial therapy, immune regulation, and improved vascularization into structural bone repair (Cao Z. et al., 2024; Li et al., 2025d; Graupe et al., 2025). Common osteogenic components include BMP-2, BMP-7, nano-hydroxyapatite, bioactive glass, Ca/P, Mg, Sr, Si, and BMSC-derived exosomes. These components can promote BMSC differentiation toward the osteogenic lineage and enhance ALP activity, collagen deposition, and mineralization capacity (Cao Z. et al., 2024; Xu et al., 2024; Graupe et al., 2025; Ling et al., 2025). If persistent infection, excessive inflammation, or insufficient vascular reconstruction remains, even materials with strong osteoinductive capacity may fail to achieve stable bone regeneration because of inadequate cell recruitment, poor blood supply, and enhanced osteoclast activity (Shi et al., 2025; Xu J. et al., 2023; Li et al., 2025d). Thus, vascular and osteogenic modules should be evaluated according to whether they restore functional perfusion and stable bone formation under sustained infection control, rather than according to isolated angiogenic or osteogenic markers (Figure 3; Table 2).

**FIGURE 3:**
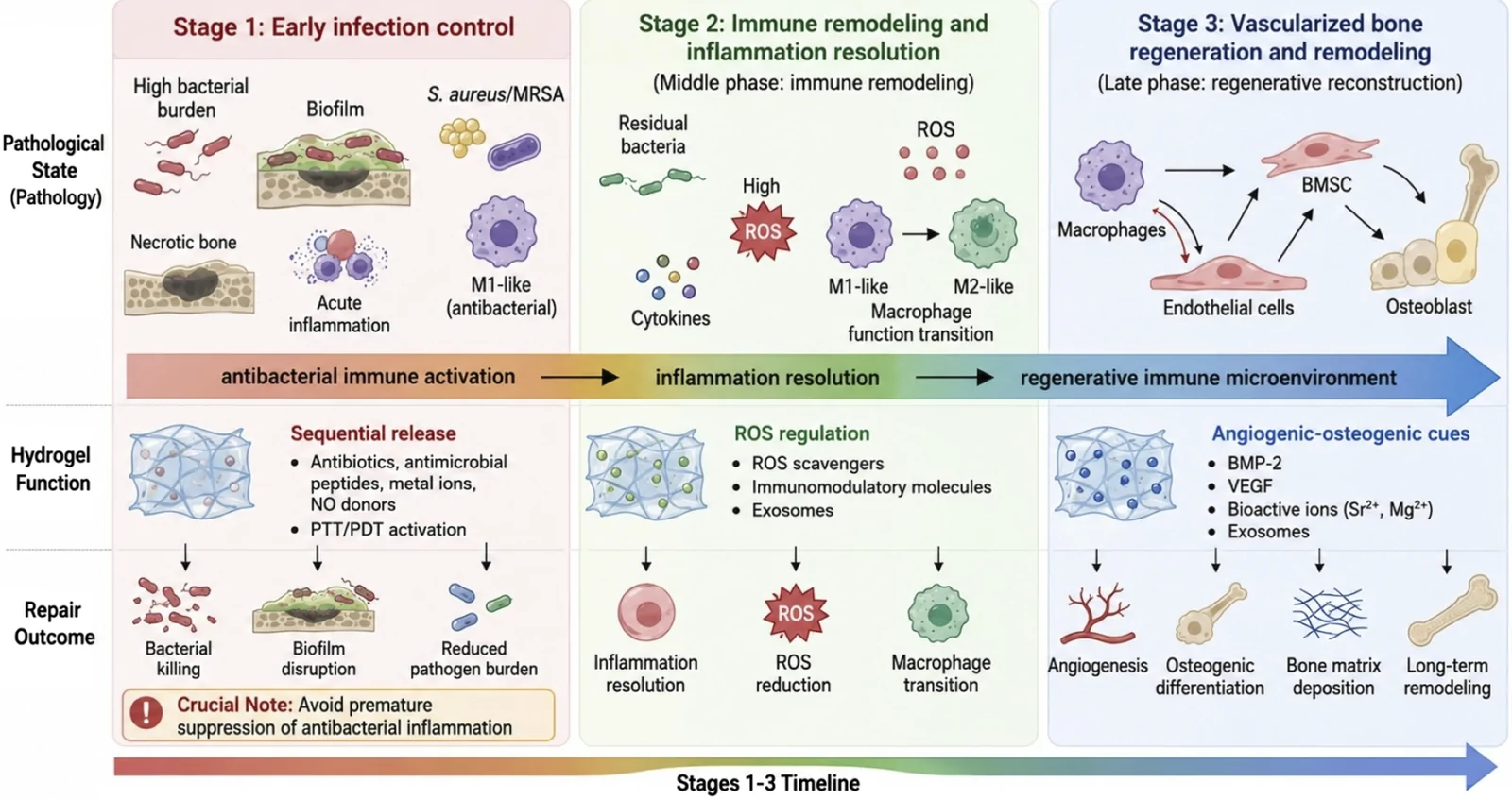
Stage-specific therapeutic logic of adaptive hydrogels: from pathogen clearance to immune remodeling and regenerative reconstruction.

**TABLE 2:** Translational assessment of representative hydrogel platforms for infected bone defect repair.

Hydrogel platform	Evidence maturity	Clinical compatibility	Complexity	Translational position	Critical next step
Single-cue responsive hydrogels	Mixed preclinical evidence: selected infected-defect and extrapolated studies	Suitable for local delivery after debridement	Low–moderate	Developing preclinical	Validate trigger thresholds in chronic infection
ROS-remodeling hydrogels	Extrapolated, early preclinical evidence: mainly inflammatory and sterile-bone models	Compatible with local implantation	Moderate	Early–developing preclinical	Confirm that ROS control does not impair bacterial clearance
Enzyme-responsive hydrogels	Mixed preclinical evidence: direct infected-defect and adjacent-model studies	Suitable for lesion-triggered release	Moderate	Developing preclinical	Validate across pathogens and disease stages
Biofilm-targeted hydrogels	Mixed preclinical evidence: strong *in vitro* data but limited infected-defect validation	Useful for dead-space and implant-surface treatment	Moderate	Developing preclinical	Test mature biofilms, recurrence, and resistance
PTT/PDT hydrogels	Partially relevant, early preclinical evidence: mainly biofilm and infected-wound models	Mainly suitable for accessible or exposed lesions	High	Early preclinical	Evaluate deep-lesion irradiation, dosimetry, and repeated-treatment safety
Multi-responsive/sequential platforms	Mixed, early preclinical evidence: selected direct infected-defect and extrapolated animal studies	Matches staged repair but may complicate clinical use	High	Early preclinical	Demonstrate superiority over simpler systems
Hydrogel–rigid scaffold composites	Extrapolated, developing preclinical evidence: mainly sterile bone-defect models	More suitable for large or load-bearing defects	Moderate–high	Developing preclinical	Conduct infected large-animal and mechanical validation
Theranostic/feedback systems	Partially relevant, exploratory evidence: infected-wound sensing without direct infected-defect validation	Potential for postoperative monitoring	Very high	Exploratory preclinical	Validate deep-tissue sensing and treatment feedback

Evidence was classified according to the original disease model, infection status, material format, and evaluated outcomes. “Direct evidence” refers to hydrogel studies conducted in infected bone defect or osteomyelitis models that evaluated infection control together with bone regeneration. “Partially relevant evidence” refers to biofilm, implant-associated infection, periodontitis, or infected soft-tissue wound studies that did not fully reproduce an infected bone defect. “Extrapolated evidence” refers to studies involving sterile bone defects, osteoarthritis, osteoporosis, diabetic or burn wounds, coatings, alloys, nanoparticles, or other non-hydrogel systems. “Mixed evidence” indicates that the supporting literature includes more than one of these categories.

## 6 Evaluation systems, translational challenges, and future directions: from functional stacking to clinically problem-driven adaptive platforms

Evaluation should determine whether hydrogel activation produces coordinated microbiological, immunological, vascular, and structural improvement within the same disease-relevant model. Unlike ordinary bone defects, infected bone defects represent a pathological ecosystem shaped by bacteria, biofilms, necrotic bone, dysregulated immune responses, and impaired regenerative capacity. Therefore, the evaluation of these materials should not be limited to a single endpoint. Instead, it should determine whether the material truly remodels the infected microenvironment and drives the repair process from a chronically destructive state toward a regenerative state.

### 6.1 Evaluation logic: from single-function validation to a full-process therapeutic chain

At present, many hydrogel studies still use short-term antibacterial efficiency, cytocompatibility, or new bone formation area as the main evidence of efficacy. However, these indicators usually demonstrate only that the material possesses a certain local function; they do not fully prove that the material can address the composite pathological problem of infected bone defects. A more rational evaluation framework should be built around a complete therapeutic chain. First, can the material be correctly activated within the infected lesion? Second, after activation, can its antibacterial action effectively reduce bacterial burden and disrupt biofilms? Third, after infection pressure decreases, can the local immune response shift from sustained pro-inflammatory activation toward inflammation resolution and tissue repair? Fourth, once inflammation is controlled, can the material further promote vascularization, osteogenic differentiation, and bone matrix reconstruction? Fifth, is the repair outcome stable in the long term, without infection recurrence, abnormal bone resorption, or material-related toxicity?

Accordingly, the evaluation of adaptive hydrogels should not focus merely on whether the material is responsive, but on whether the response occurs in response to the correct pathological signal, within the correct therapeutic window, and at the appropriate stage of treatment. For example, pH-, ROS-, enzyme-, hypoxia-, or photothermal responsiveness does not automatically equate to precision therapy. If a material only demonstrates release behavior in an *in vitro* buffer system but lacks validation in a real infected lesion, its responsiveness remains largely conceptual. A truly meaningful responsive design should match the stage-specific requirements of infected bone defects: prioritizing bacterial and biofilm clearance in the early stage, limiting excessive inflammation and oxidative injury in the intermediate stage, and supporting vascularization and bone regeneration in the later stage. In other words, when evaluating adaptive hydrogels, the key is not to prove that they have many functions, but to demonstrate that these functions have a rational temporal sequence and pathological adaptability.

Antibacterial evaluation should likewise shift from bactericidal efficiency alone to the quality of infection control. In infected bone defects, the most difficult problem is not short-term killing of planktonic bacteria, but biofilms formed on necrotic bone, implant surfaces, and material interfaces, as well as the risk of recurrence caused by residual bacteria after treatment. Therefore, a convincing study of antibacterial hydrogels should simultaneously examine mature biofilm disruption, residual bacterial regrowth, drug-resistance risk, and host tissue protection. Excessively strong antibacterial stimulation, metal ion release, or photothermal effects may improve short-term bacterial killing, but they may also damage osteoblasts, endothelial cells, and local immune cells, thereby compromising the subsequent regenerative microenvironment. The goal of infected bone defect treatment is not to maximize bacterial killing at all costs, but to effectively control infection while preserving the capacity for tissue repair.

Osteoimmune evaluation should also avoid oversimplification. In infected bone defects, macrophage regulation is not simply a matter of promoting M2 polarization as early as possible, nor does suppression of inflammation necessarily indicate a favorable repair response. During the early stage of infection, sufficient pro-inflammatory and phagocytic antibacterial activity is required; otherwise, pathogen clearance may be inadequate. After bacterial burden decreases, however, persistent pro-inflammatory activation can promote osteoclast activation, suppress osteogenesis, and delay vascularization. Therefore, material evaluation should emphasize dynamic immune regulation rather than static phenotype conversion. An ideal hydrogel should support host pathogen clearance in the early stage and then promote inflammation resolution, angiogenesis, and bone reconstruction in the middle and late stages. This evaluation logic is more consistent with the pathological process of infected bone defects than simply measuring M1/M2 markers at a single time point.

Evaluation of bone regeneration should be based on the premise that infection has been effectively controlled. For infected bone defects, observing new bone formation alone is insufficient, because local infection relapse, residual chronic inflammation, or enhanced bone resorption may still lead to late-stage repair failure. More reasonable criteria should include whether new bone forms in an environment without obvious infection recurrence, whether vascularization is sufficient to support the repair of large-volume bone defects, whether osteogenesis and osteoclastogenesis have been rebalanced, and whether the new bone structure has long-term stability and mechanical significance. Only when infection control, immune remodeling, vascularization, and bone formation are evaluated along the same timeline can we truly determine whether adaptive hydrogels have achieved continuous repair of infected bone defects (Zhang et al., 2025b). Evaluation of lesion-state-dependent switching should establish three linked relationships. First, the target cue or biomarker should be quantified within the same infected bone defect model. Second, changes in that signal should be correlated with material activation, cargo release, or functional switching. Third, the resulting switch should be associated with improved microbiological, inflammatory, vascular, or regenerative outcomes without increasing recurrence or host-tissue injury. Studies should prospectively define candidate transition rules rather than retrospectively assigning treatment phases according to time after implantation. Relevant measurements may include viable bacterial counts, biofilm burden, bacterial products, local pH, ROS intensity, bacterial or host enzyme activity, oxygen tension, cytokine profiles, material degradation state, vascular perfusion, and bone-regeneration markers.

### 6.2 Model construction: from convenient validation to clinically relevant evaluation

Animal models determine the credibility of material evaluation. At present, many studies use small-animal acute infection models, which are convenient to establish, require relatively short experimental periods, and are suitable for early proof-of-concept validation. However, these models remain distant from clinical scenarios such as chronic osteomyelitis, implant-associated infection, drug-resistant bacterial infection, and complex post-traumatic infection. In real clinical practice, infected bone defects are often accompanied by residual necrotic bone, insufficient blood supply, biofilm formation, polymicrobial infection, repeated debridement, and the need for internal fixation, whereas these factors are frequently weakened or absent in simplified animal models (Reizner et al., 2014). Therefore, future studies should select models according to the core function of the material rather than using a single model to validate all types of hydrogel systems. For photoactivated hydrogel systems, model construction should also reproduce clinically relevant optical and anatomical barriers. Direct irradiation of a superficially exposed defect in a small animal may substantially overestimate therapeutic efficacy. Future studies should report the depth of the target lesion, thickness and type of overlying tissues, irradiation angle and distance, presence of necrotic bone or internal fixation, and whether the defect is surgically exposed during treatment. Temperature should be monitored not only at the skin surface but also within the defect and in adjacent healthy tissues whenever technically feasible. Repeated-treatment protocols should be evaluated to determine whether antibacterial efficacy can be maintained without cumulative thermal, photochemical, vascular, or neurological injury. For systems intended for deep or load-bearing infections, large-animal or clinically representative implant-associated models may provide more meaningful evidence than superficial small-animal defects.

If a material mainly emphasizes antibiofilm capacity, mature biofilm models or implant-associated infection models should be prioritized. If the material mainly focuses on bone regeneration, the defect should be sufficiently critical in size to avoid spontaneous healing that may mask the material effect. If the material claims immunomodulatory activity, an adequate observation window should be included to capture immune-state transitions after bacterial burden decreases. If the material is intended for clinically complex infections, future studies should gradually introduce models involving chronic infection, drug-resistant bacteria, polymicrobial infection, or underlying disease conditions. For large segmental bone defects and infections at load-bearing sites, mouse or rat models alone are often insufficient to evaluate mechanical adaptability and surgical operability. In this context, large-animal models, internal fixation-associated infection models, and post-debridement reconstruction models may provide greater translational value (Bottagisio et al., 2019).

Greater standardization is also needed in this field. Current studies vary considerably in bacterial strain selection, inoculation dose, infection duration, defect size, intervention timing, follow-up period, and primary endpoints, making cross-study comparison difficult. Future studies should report the modeling process, infection diagnostic criteria, randomization and blinding methods, sample size justification, animal attrition, and primary outcome measures more clearly (Meroni et al., 2022; Sert et al., 2020). For hydrogel studies, it is also important to clarify the relationship between material implantation timing and clinical workflow: whether the hydrogel is implanted prophylactically before infection formation or therapeutically after debridement; and whether it is intended for acute infection, chronic osteomyelitis, or recurrent infection. Only when the modeling context is clearly defined can the therapeutic effect of a material be meaningfully interpreted.

### 6.3 Translational challenges: multifunctionality does not necessarily mean clinical applicability

The translational bottleneck of adaptive hydrogels lies not only in whether their therapeutic efficacy is sufficiently strong, but more importantly in the tension among material complexity, safety, manufacturing consistency, and clinical operability. Many laboratory-designed hydrogels have become increasingly complex, often incorporating natural polymers, synthetic polymers, antibiotics, metal ions, nanomaterials, photothermal agents, growth factors, exosomes, or nucleic acid delivery systems within a single platform. Although such complex designs help demonstrate multifunctionality, they also increase compositional uncertainty, batch-to-batch variability, sterilization difficulty, long-term metabolic risk, and regulatory complexity. Multi-component systems also introduce risks that cannot be predicted by evaluating each component separately. Chemical interactions among polymers, antibiotics, metal ions, nanomaterials, proteins, or nucleic acids may alter crosslinking efficiency, degradation behavior, drug stability, and release kinetics. Components designed for different therapeutic phases may be released simultaneously or in an unintended order, resulting in subtherapeutic exposure, burst release, cumulative toxicity, or loss of stage-specific regulation. Biological effects may also conflict: antibacterial ROS, metal ions, and photothermal agents can damage bacteria but may simultaneously impair osteoblasts, endothelial cells, and immune cells, whereas excessive antioxidant or anti-inflammatory activity may compromise host defense. Increasing the number of components further complicates sterilization, storage, quality control, and regulatory evaluation. Heat, irradiation, or chemical sterilization may alter polymer networks or reduce the activity of growth factors, exosomes, and nucleic acid therapeutics. Batch-to-batch variation may arise from differences in natural polymers, nanoparticles, biological products, or loading efficiency. Moreover, when several active components are incorporated into one system, it becomes difficult to determine which component is responsible for efficacy or toxicity. Therefore, each additional module should be justified by a clearly defined clinical need and should demonstrate measurable benefit over a simpler formulation. For clinical translation, more functions do not necessarily mean better materials. Instead, under a clearly defined clinical problem, material design should be as simple, stable, and controllable as possible while achieving the key therapeutic objectives. The translational maturity of photoactivated hydrogels should therefore be interpreted cautiously. High bactericidal rates in planktonic bacterial cultures or thin biofilm models primarily demonstrate proof-of-concept photothermal or photodynamic activity. Similarly, successful treatment of superficial small-animal defects under direct irradiation does not establish efficacy in deep, chronically infected, or mechanically complex human bone defects. Before clinical translation can be considered, these systems require standardized dosimetry, clinically relevant penetration testing, real-time thermal monitoring, repeated-treatment safety evaluation, validation in chronic and implant-associated infection models, and assessment of compatibility with debridement, fixation, soft-tissue reconstruction, and postoperative care. At present, the most plausible clinical scenarios may be surgically exposed lesions, postoperative dead spaces, or defects in which optical fibers or other controlled light-delivery devices can be safely introduced.

Safety is another issue that must be considered at an early stage of material design. Repair of infected bone defects often requires long-term implantation or local retention of materials. Therefore, evaluation should not be limited to short-term cytocompatibility, but should also include degradation products, local accumulation, chronic inflammation, ectopic or abnormal bone formation, immunogenicity, and potential systemic toxicity. Antibiotic-loaded systems should be carefully assessed for the risk of drug resistance caused by long-term low-dose release. For metal ions and inorganic nanomaterials, the therapeutic window between effective and toxic doses must be clearly defined. Photothermal and photodynamic systems need to consider light penetration, temperature control, and the feasibility of repeated treatment in deep bone tissues. Systems containing growth factors, exosomes, or nucleic acid therapeutics face additional challenges related to stability, batch consistency, and biosafety. If these issues are not fully considered during early material design, even promising *in vivo* results may be insufficient to support further preclinical development.

Mechanical adaptability is also a practical challenge for translating hydrogels in infected bone defect repair. Hydrogels possess favorable injectability, local retention, and microenvironment-modulating capacity, but most hydrogels alone cannot provide the structural support required for load-bearing bone defects. For small, non-load-bearing defects or post-debridement dead-space filling, hydrogels can serve as local delivery and immunomodulatory platforms. However, for large segmental defects or infections at load-bearing sites, hydrogels may be more suitable as part of a composite system, in combination with 3D-printed scaffolds, inorganic bone scaffolds, bone cement, metallic internal fixation, or external fixation techniques. A more clinically promising direction may not be the development of a single “all-in-one hydrogel,” but rather a composite therapeutic system in which rigid scaffolds provide structural support while soft hydrogels regulate the infected microenvironment.

In addition, material design must be embedded within real clinical workflows. Treatment of infected bone defects usually involves radical debridement, dead-space management, local or systemic antibiotic therapy, stable fixation, soft tissue coverage, and staged bone reconstruction. If hydrogels are to enter clinical practice, they must answer several practical questions: Can they be directly implanted after debridement? Do they interfere with subsequent fixation or bone grafting? Can they be combined with commonly used antibiotics or bone cement? Are they convenient for storage, transportation, and intraoperative handling? Can the dosage or administration frequency be adjusted according to infection status? Only when material innovation is compatible with clinical pathways can it move from a laboratory concept toward a usable therapeutic tool.

### 6.4 Future directions: precision adaptation, minimally sufficient design, and theranostic integration

Future adaptive hydrogel research should shift from multifunctional stacking and fixed release schedules toward lesion-state-driven regulation. Key priorities include defining clinically meaningful sensing thresholds, linking detected signals to specific material responses, demonstrating superiority over simpler formulations, and maintaining compatibility with manufacturing and surgical workflows. Because bacterial persistence, inflammation, hypoxia, and attempted regeneration may coexist, future systems should rebalance therapeutic functions according to validated combinations of microbiological, inflammatory, metabolic, and material-state signals. “Minimally sufficient design” may become an important principle for future translational research. Rather than constructing multifunctional platforms with overly complex compositions and dispersed mechanisms, it may be more valuable to design modular systems that are verifiable, manufacturable, and regulatable around a clearly defined clinical problem. For example, in cases of residual infection after debridement, antibiofilm and local antibacterial modules should be prioritized. For regenerative failure after chronic infection, immune remodeling and vascularization modules may be emphasized. For large segmental bone defects, hydrogels should be integrated with mechanically supportive scaffolds rather than being expected to perform all functions independently. Such modular design is beneficial not only for mechanistic clarification but also for subsequent quality control and clinical translation.

Future studies should also strengthen mechanistic stratification and precision evaluation. Single-cell sequencing, spatial transcriptomics, metabolomics, multiplex immunofluorescence, and *in vivo* imaging can be used to dissect the dynamic interactions among bacteria, immune cells, osteoblasts, osteoclasts, and vascular endothelial cells within infected lesions after hydrogel treatment. These approaches may help answer one of the most critical questions in this field: does the material promote bone repair indirectly by simply reducing bacterial burden, or does it actively remodel the osteoimmune microenvironment and drive regeneration? Only by linking material effects with pathological mechanisms can adaptive hydrogel research move from “phenomenological efficacy” toward “mechanistic clarity.”

Theranostic integration is another promising direction. During the treatment of infected bone defects, clinicians need not only materials that release therapeutic agents, but also tools to determine whether infection has been controlled, whether inflammation is resolving, and whether bone regeneration has been successfully initiated. Therefore, future hydrogels may integrate monitoring functions for pH, ROS, enzymatic activity, oxygen concentration, temperature, or bacterial metabolic signals, enabling visualization of the therapeutic process and feedback-regulated intervention. Particularly for chronic osteomyelitis, implant-associated infection, and recurrent infection, hydrogel platforms capable of sensing lesion status and providing feedback on treatment progression may better meet clinical needs than one-time release systems. However, sensing alone does not establish feedback regulation. A genuinely adaptive platform should connect a validated lesion-state signal with a defined material response and demonstrate that the resulting switch improves treatment outcomes. Determining clinically meaningful signal combinations and transition thresholds remains a major research priority.

Overall, the future value of adaptive hydrogels in infected bone defect repair should not be understood merely as an improvement of a new drug-loading material, but rather as a local microenvironment remodeling strategy. Their true goal is to integrate antibacterial therapy, immune regulation, vascularization, and bone regeneration into a continuous, controllable, and evaluable repair process. Hydrogel platforms with genuine translational potential should have clearly defined clinical indications, simple and controllable material compositions, stable and reproducible fabrication processes, interpretable mechanisms of action, reliable long-term safety, and modes of use that are compatible with surgical workflows. Only when these conditions are simultaneously met can adaptive hydrogels move beyond multifunctional laboratory concepts and toward clinical application in the precision repair of infected bone defects.
